# CTCF DNA-binding domain undergoes dynamic and selective protein–protein interactions

**DOI:** 10.1016/j.isci.2022.105011

**Published:** 2022-08-24

**Authors:** Rong Zhou, Kai Tian, Jie Huang, Wenjia Duan, Hongye Fu, Ying Feng, Hui Wang, Yongpeng Jiang, Yuanjun Li, Rui Wang, Jiazhi Hu, Hanhui Ma, Zhi Qi, Xiong Ji

**Affiliations:** 1 Key Laboratory of Cell Proliferation and Differentiation of the Ministry of Education, School of Life Sciences, Peking-Tsinghua Center for Life Sciences, Peking University, Beijing 100871, China; 2 Center for Quantitative Biology, Peking-Tsinghua Center for Life Sciences, Academy for Advanced Interdisciplinary Studies, Peking University, Beijing 100871, China; 3 School of Life Science and Technology, ShanghaiTech University, Shanghai 201210, China

**Keywords:** Biological sciences, Molecular biology, Molecular interaction

## Abstract

CTCF is a predominant insulator protein required for three-dimensional chromatin organization. However, the roles of its insulation of enhancers in a 3D nuclear organization have not been fully explained. Here, we found that the CTCF DNA-binding domain (DBD) forms dynamic self-interacting clusters. Strikingly, CTCF DBD clusters were found to incorporate other insulator proteins but are not coenriched with transcriptional activators in the nucleus. This property is not observed in other domains of CTCF or the DBDs of other transcription factors. Moreover, endogenous CTCF shows a phenotype consistent with the DBD by forming small protein clusters and interacting with CTCF motif arrays that have fewer transcriptional activators bound. Our results reveal an interesting phenomenon in which CTCF DBD interacts with insulator proteins and selectively localizes to nuclear positions with lower concentrations of transcriptional activators, providing insights into the insulation function of CTCF.

## Highlights

• The CTCF DNA-binding domain forms protein clusters *in vivo* and *in vitro*
• CTCF DBD clusters colocalize with insulator proteins but not with activators
• Arginine residues of CTCF DBD are frequently mutated in cancers
• Multiple transcription factor DBDs form protein clusters

## Graphical abstract

**Figure:**
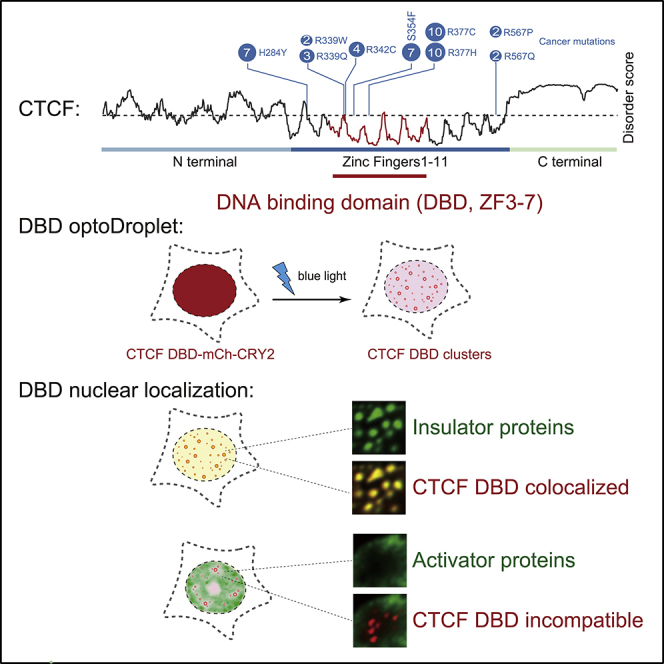

## Introduction

Insulators are cis-regulatory elements that play a central role in regulating cell-type-specific gene expression during development and disease (Flavahan et al., 2016; Herold et al., 2012). The insulation function blocks enhancer-activating promoters (Heger and Wiehe, 2014; Raab and Kamakaka, 2010; Recillas-Targa et al., 2002), and many protein factors, such as CTCF, BRD2, CHD8, and DDX5, have been reported to bind insulator elements and perform insulation functions (Bell et al., 1999; Hsu et al., 2017; Ishihara et al., 2006; Yao et al., 2010). Previous studies have investigated the DNA sequences that are required for CTCF-mediated insulation (Guo et al., 2015; Huang et al., 2021). CTCF is known to form loops with Cohesin by loop extrusion. Enhancers localize within CTCF loops and cannot activate genes outside CTCF loops (Dowen et al., 2014; Ji et al., 2016; Sun et al., 2019). However, it is still difficult to understand how these CTCF loops physically block enhancers to activate gene expression outside loops in the three-dimensional nuclear organization. CTCF binds to CCCTC DNA motifs in the genome and interacts with insulator proteins, and the roles of CTCF protein domains in insulation have not been fully explained in mammals (Bell et al., 1999; Nora et al., 2017; Zuin et al., 2014).

CTCF comprises an *N*-terminal domain (NTD), 11 zinc fingers, and *C*-terminal intrinsically disordered regions (IDRs; Ghirlando and Felsenfeld, 2016; Merkenschlager and Odom, 2013; Ong and Corces, 2014; Ohlsson et al., 2001; Vietri Rudan and Hadjur, 2015; Zlatanova and Caiafa, 2009). The NTD of CTCF interacts with Cohesin in chromatin to organize 3D chromatin structures through loop extrusion (Li et al., 2020; Nora et al., 2020; Pugacheva et al., 2020). The zinc finger 1 and 10 and *C*-terminal domains of CTCF show RNA-binding activities (Hansen et al., 2019; Saldana-Meyer et al., 2014, 2019), and zinc fingers 3–7 of CTCF constitute the DNA-binding domain (DBD), which directly interacts with DNA (Hashimoto et al., 2017; Yin et al., 2017). The RNA-binding domain (RBD) mediates CTCF self-interactions and is essential for CTCF-organized 3D chromatin structures. Although each domain of CTCF has been intensively studied in a 3D chromatin organization, it is still difficult to understand how these CTCF loops block enhancer functions in the three-dimensional nucleus.

By taking advantage of the optoDroplet system to detect weak, dynamic, and transient protein–protein interactions, we found that the CTCF DBD both interacts with itself and selectively interacts with other insulator proteins but is not coenriched with transcriptional activators in the nucleus, which is a finding distinct from the generally assumed role of CTCF in DNA binding. Similar properties were not observed for other domains of CTCF or the DBDs of other transcription factors. Super-resolution imaging and bioinformatic and insulator reporter assays showed that endogenous CTCF forms small protein clusters and that its binding sites in the genome contain CTCF motif arrays that are associated with a low abundance of transcriptional activators and are positively correlated with insulator activity. Overall, we provide experimental evidence to help establish a framework accounting for the insulation functions of CTCF.

## Results

### Examination of dynamic CTCF DBD self-interactions with the optoDroplet system and *in vitro*-purified proteins

**Figure 1:**
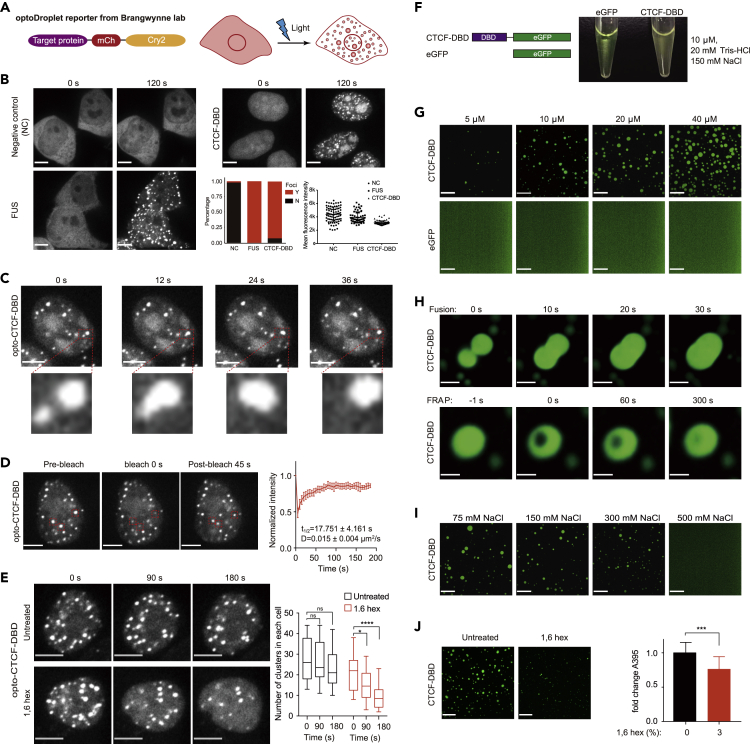
The CTCF DBD undergoes self-interaction *in vitro* and in cells with optoDroplet (A) Schematic illustration of the optoDroplet reporter (left) and blue light-induced target protein domain clustering in live cells (right). (B) Images of HEK293T cells expressing mCherry-Cry2, FUSN, or the DBD of CTCF fused to mCherry-Cry2 (opto). Representative images of light-activated cells are shown. Fluorescent proteins expressed at similar levels were activated under identical conditions. The percentages of cells forming protein clusters are shown in the bar graphs. Y indicates observed clusters; N indicates that no clusters were observed. Fluorescent signals for the protein expression level used in the optoDroplet assay are shown at the bottom right. Data are represented as the mean ± SD. At least *n* = 52 cells were used for the calculation. Scale bars, 5 μm. (C) A CTCF DBD-opto cluster fusion event is shown, with a higher-resolution image below. Scale bars, 5 μm. (D) Representative images of an FRAP experiment with CTCF DBD-opto in HEK293T cells. Red boxes indicate bleached clusters (left). Quantitative analyses of FRAP data of a CTCF DBD-opto cluster (right). A bleaching event occurred at *t* = 0 s. Data are represented as the mean ± SD (*n* = 5). Scale bars, 5 μm, Apparent D: apparent diffusion coefficient; *t*1/2: half-time of recovery. (E) Representative images of CTCF DBD-opto in HEK293T cells treated with 3% 1,6-hexanediol for 90 and 180 s (left). Box plot illustration of the fold change in the number of CTCF DBD-opto clusters under 1,6-hexanediol treatment (right). *n* = 20 in the control and *n* = 20 in the 1,6-hexanediol treatment group were used for calculation. *p*-values were calculated using the unpaired two-tailed Student’s *t*-test (ns not significant, ∗<0.05, ∗∗∗∗<0.0001). Scale bars, 5 μm. (F) Schematic illustration of the recombinant eGFP and CTCF DBD-eGFP used here (left). Turbidity analyses of the CTCF DBD and eGFP in buffer (20-mM Tris-HCl, 150 mM NaCl) at a concentration of 10 μM at the room temperature (right). (G) Representative images of droplet formation in the presence of different protein concentrations: CTCF DBD-eGFP or EGFP (bottom). Scale bars, 24 μm. (H) Representative images of droplet fusion events and photobleaching recovery at the indicated time points. Scale bars, 5 μm (Fusion)/2.5 μm (FRAP). (I) Representative images of CTCF DBD-eGFP droplet formation in the presence of different concentrations of NaCl. Scale bars, 24 μm. (J) Representative images of CTCF DBD droplet formation after treatment with 1,6-hexanediol (left) and absorbance analyses at 395 nm (A395) of CTCF DBD proteins in phase separation buffer (right). *p*-values were calculated using an unpaired two-tailed Student’s *t*-test (∗∗∗<0.001). Scale bars, 24 μm. Data are represented as the mean ± SD.

Transcription factors comprise low-complexity domains (LCDs) and DBDs. Previous studies have shown that transcription factors can form local concentrated hubs through weak, transitory, dynamic LCD-LCD interactions (Chong et al., 2018). We sought to investigate whether the DBD also mediates dynamic protein–protein interactions, which may provide different functional aspects of transcription factors. To this end, we performed an optoDroplet assay with CTCF DBD (CTCF zinc finger 3–7), which is reported to interact directly with DNA (Hashimoto et al., 2017; Yin et al., 2017). Note: ZF3-7 interacts directly with DNA according to the crystal structure *in vitro*, and other zinc fingers also contribute to the DNA binding of full-length CTCF in cells (Nakahashi et al., 2013; Saldana-Meyer et al., 2019; Soochit et al., 2021). The optoDroplet system is a previously developed light-inducible reporter system used to determine which protein domains are able to self-interact to form protein clusters in mammalian cells (Shin et al., 2017; Figure 1A). Under similar protein expression levels to NC and FUS, the optoDroplet experiments suggested that the CTCF DBD formed protein clusters, which were recognized as individual spherical, droplet-like objects, indicating the self-interaction of the CTCF DBD in cells (Figure 1B). The opto-CTCF-DBD is roughly similar to the concentrations of endogenous CTCF reported previously (Cattoglio et al., 2019; Holzmann et al., 2019; Figure S1A). We also found that the optoDroplet CTCF NTD did not form clusters and that the *C*-terminal IDR and RBD could form self-interacting protein clusters (Saldana-Meyer et al., 2014; Figure S1B), consistent with previous findings demonstrating that disruption of the RBD affects CTCF clustering (Hansen et al., 2019; Saldana-Meyer et al., 2019). The IDR and RBD domains show the potential to form protein clusters (Hansen et al., 2019, 2020; Saldana-Meyer et al., 2014, 2019), but the formation of protein clusters by the CTCF DBD was unexpected. Thus, we believe that the CTCF DBD that forms protein clusters is not reported, and we focused on the CTCF DBD in the rest of this study.

We next determined whether the optoDroplet CTCF DBD *per se* could form biomolecular condensates in cells. The features of condensates typically include a capacity for fusion, dynamic exchange with the local environment, and sensitivity to the disruption of hydrophobic interactions (Alberti et al., 2019; Shin and Brangwynne, 2017). Taking advantage of the optoDroplet system again, we monitored the fusion of CTCF DBD clusters in detail (Figure 1C and Video S1). Our fluorescence recovery after photobleaching (FRAP) experiments indicated that the signals of the CTCF DBD clusters recovered within seconds upon photobleaching, similar to the recovery of previously reported condensates (Figure 1D and Video S2). The FRAP recovery of the optoDroplet CTCF DBD was incomplete at 60 s and did not fully recover after 200 s. Moreover, the CTCF DBD clusters were sensitive to 1,6-hexanediol, as treatment with this compound caused their dissolution within approximately 3 min (Figure 1E and Videos S3a and S3b). Collectively, these results indicate that the optoDroplet CTCF DBD exhibits condensate-like characteristics.

Video S1. Formation of opto-CTCF DBD droplets in HEK293T cells upon stimulation with blue light, related to Figure 1

Video S2. FRAP of opto-CTCF DBD droplets formed in HEK293T cells, related to Figure 1

Video S3a. A time-lapse movie of opto-CTCF DBD droplets after blue light activation under untreated conditions, related to Figure 1

Video S3b. Time-lapse movie of opto-CTCF DBD droplets upon treatment with 3% 1,6-hexanediol, related to Figure 1

We examined whether the *in vitro*-purified CTCF DBD proteins exhibited biomolecular condensate features. We expressed and purified recombinant control eGFP and CTCF-DBD-eGFP fusion proteins to facilitate the detailed characterization of the cluster-forming behavior of the CTCF DBD (Figures S1C–S1E). The CTCF-RBD formed protein clusters, whereas the CTCF-NTD did not form clusters using a similar experimental system (Figure S1F). Notably, the purified CTCF DBD fusion protein became opaque in buffer (20-mM Tris-HCl, 150-mM NaCl) at a concentration of 10 μM at the room temperature, but purified EGFP did not (Figure 1F). Fluorescence microscopy analyses indicated that the CTCF DBD protein formed spherical clusters in a concentration-dependent manner, whereas EGFP did not (Figure 1G and Video S4). Moreover, the CTCF DBD clusters could fuse and recover rapidly after photobleaching (Figure 1H and Video S5) and were highly sensitive to the high-salt and 1,6-hexanediol treatments used to assess the phase separation behavior of the proteins (Figures 1I and 1J). These results collectively suggested that the *in vitro* protein–protein interactions of the CTCF DBD are weak, dynamic, and transient, which is reminiscent of previously described LCD–LCD interactions (Chong et al., 2018).

Video S4. Time-lapse imaging of 10-μM CTCF DBD-EGFP phase separation, related to Figure 1

Video S5. FRAP and fusion events of CTCF DBD-EGFP droplets in vitro, related to Figure 1

### CTCF DBD optoDroplets selectively interact with insulator proteins and tend to avoid nuclear positions with a high abundance of transcriptional activators

As the protein domains of CTCF can be efficiently clustered in cells with the optoDroplet system, we next sought to investigate how the domains of CTCF contribute to its protein interactions. We first cotransfected the CTCF DBD, RBD, NTD, or IDR optoDroplet plasmid with several eGFP-tagged transcriptional regulator plasmids. Then, the cells were exposed to blue light for up to 3 min to induce them to form relatively stable protein clusters, and the relative positions of the opto fusions and appropriately expressed eGFP fusions were analyzed. These experiments allowed us to examine many combinations of these sequences easily.

**Figure 2:**
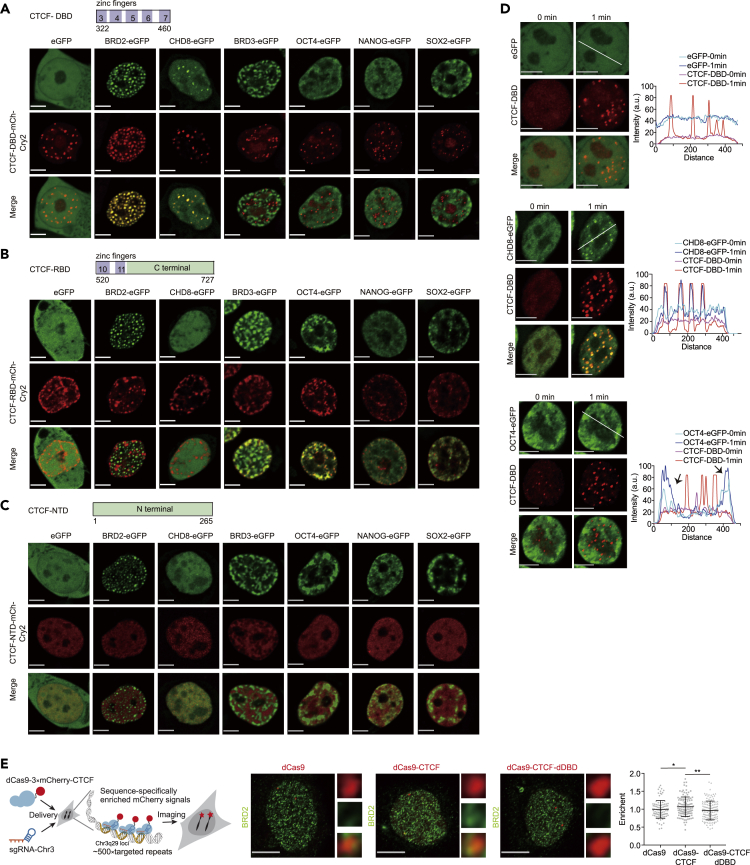
The CTCF DBD selectively interacts with insulator proteins but avoids transcriptional activators (A) Representative images of HEK293T cells expressing CTCF-DBD-mCh-Cry2 with eGFP, BRD2-eGFP, CHD8-eGFP, BRD3-eGFP, OCT4-eGFP, NANOG-eGFP, and SOX2-eGFP. Representative images of blue light-activated cells are shown. Scale bars, 5 μm. (B) Representative images are shown the same as A but for CTCF-RBD-mCh-Cry2. Scale bars, 5 μm. (C) Representative images are shown the same as A but for CTCF-NTD-mCh-Cry2. Scale bars, 5 μm. (D) Fluorescence images of HEK293T cells expressing CTCF-DBD-mCh-Cry2 with eGFP (top), CHD8-eGFP (middle), and OCT4-eGFP (bottom) before and after 1 min of stimulation with blue light. Scale bars, 5 μm. The fluorescence intensity profiles at different positions in the CTCF DBD clusters before and after stimulation with light in the 488- and 561-nm channels (bottom) are indicated by a white line. Arrowheads indicate regions with increased signals after blue light stimulation. We observed that 0/19 CTCF DBD clusters excluded eGFP, 19/26 CTCF DBD clusters recruited CHD8-eGFP, and 5/20 CTCF DBD clusters excluded OCT4-eGFP. N/M = clusters showing exclusion or recruitment/total clusters. (E) Left panel: the experimental design for dCas9 tethering of full-length CTCF to endogenous genomic loci. The schematic diagram was edited from that in Chen et al. (2013). Middle panel: representative immunofluorescence (IF) images of BRD2 in U2OS cells expressing sgRNA-ChrC3 with dCas9, dCas9-CTCF, or dCas9-CTCF-dDBD. The right images show the magnified areas. Right panel: scatter plot illustrating the enrichment of the intensity of the BRD2 immunofluorescence signal at the indicated tethered loci. BRD2 enrichment was calculated by dividing the average BRD2 fluorescence intensities around the center of the dCas9 foci by the average BRD2 signals of the whole nucleus. At least *n* = 66 cells were used for the calculation. Data are represented as the mean ± SD. Scale bars, 10 μm. *p*-values were calculated using an unpaired two-tailed Student’s *t*-test (∗∗<0.01, ∗<0.05). Scale bars, 5 μm.

CHD8 is a chromatin remodeler that was previously reported to cooperate with CTCF in the insulation function, and the histone acetylation reader BRD2 facilitates CTCF boundary activity (Hsu et al., 2017; Ishihara et al., 2006). The results showed that the insulator proteins BRD2 and CHD8 colocalized with CTCF DBD clusters but not with the CTCF RBD, NTD, or IDR protein clusters after blue light induction (Figures 2A–2C and S2A), which is consistent with previous observations that CTCF interacts with CHD8 and BRD2 (Hsu et al., 2017; Ishihara et al., 2006). Interestingly, BRD2 clusters appear to attract CTCF-DBD, and CTCF DBD clusters seem to induce the clustering of CHD8, indicating that the behavior of BRD2 and CHD8 in relation to CTCF DBD is different (Figures 2A and 2D). The CTCF DBD clusters tended to be enriched at nuclear positions with lower concentrations of the transcriptional activators BRD3, OCT4, NANOG, and SOX2, and these active apparatuses appeared to colocalize with RBD and IDR clusters (Figures 2A–2C and S2A). GFP was distributed almost homogeneously in the nucleus with CTCF DBD clusters (Figure 2A) and served as a negative control.

To provide evidence of the protein partner interactions of the CTCF DBD, we performed live-cell imaging before and after blue light irradiation for 1 min. The results showed that the green fluorescent signals of CHD8 increased in regions within CTCF DBD optoDroplets, and the eGFP signals did not change (Figure 2D), indicating that our opto-quantification system functioned properly. The CTCF DBD optoDroplets appeared to converge at positions with a low density of OCT4 green fluorescent signals (Figure 2D). These results suggest that CTCF DBD protein clusters incorporate the insulator protein CHD8 and avoid positions with high densities of OCT4. Interestingly, not all CTCF DBD clusters occupied positions with a low density of OCT4 (Figure 2D), indicating that the avoidance behavior of the CTCF DBD is context-dependent. Moreover, we used dCas9 to tether full-length CTCF and DBD-deleted CTCF to repeated genomic regions (Chen et al., 2013; Ma et al., 2015, 2016; Wang et al., 2018), such that the tethered proteins would be visible via fluorescent imaging. The results showed that tethering full-length CTCF increased BRD2 signals in the targeted regions, whereas tethering CTCF with DBD deletion did not (Figure 2E). These results were consistent with the idea that CTCF participates in protein–protein interactions via its DBD.

### Endogenous CTCF forms small protein clusters, interacts with CTCF motif arrays in the genome, and avoids regions with a high density of transcriptional activators

**Figure 3:**
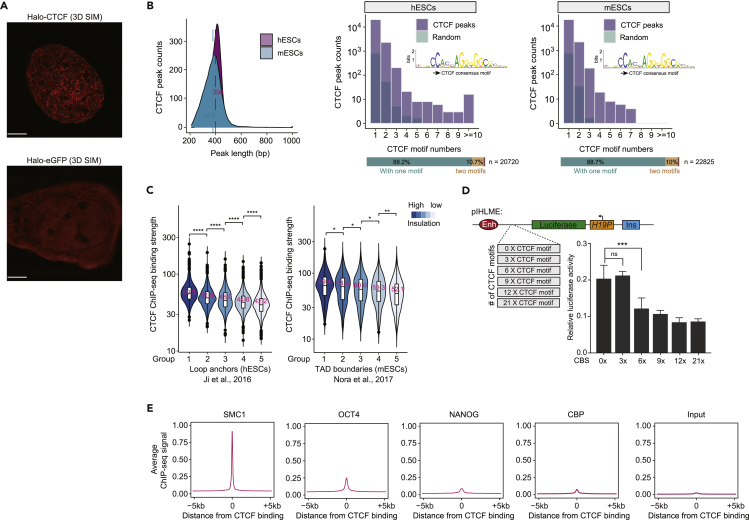
Full-length CTCF forms small protein clusters and interacts with CTCF motif arrays occupied by low densities of transcriptional activators (A) 3D SIM imaging of halo-tagged CTCF (top) or halo-eGFP (bottom) in U2OS cells. Scale bars, 5 μm. (B) Left: CTCF peak length distributions of ChIP-seq data generated from human embryonic stem cells (hESCs) and ChIP-exo data derived from mouse embryonic stem cells (mESCs). Right: histogram displaying CTCF motif occurrence in CTCF ChIP-seq peaks identified in hESCs and mESCs, with the bottom bar indicating the percentage of occurrence frequency of this motif. The light purple and cyan colors indicate CTCF peaks and randomly selected regions in the corresponding human and mouse genomes, respectively. The position weight matrix for the canonical JASPAR CTCF motif finding is shown at the middle right. (C) Left: violin plot of CTCF ChIP-Seq signals corresponding to different insulation strengths at loop anchors from cohesin ChIA-PET data in hESCs (1–5: insulation strength from high to low). The analyses of the left anchor are shown, and the right anchor behaves in the same way. Right: same as the left but for TAD anchors from Hi-C data in mESCs. (D) Schematic illustrations of the pIHLME reporter used in the luciferase assay (top). Luciferase activities of the pIHLME construct in the presence of the indicated number of CTCF motif insertions in HEK293T cells (bottom right). *p*-values were calculated using two-tailed Student’s *t*-tests (∗∗∗<0.001, ∗<0.05). Data are represented as the mean ± SD. The CTCF motif sequences are shown in Table S5. (E) Averaged ChIP-Seq signals of Cohesin (SMC1), OCT4, NANOG, CBP, and input control at CTCF-binding sites.

We next performed CTCF immunofluorescence analysis using different antibodies and fixation protocols to enhance the protein signals. The results revealed that CTCF could indeed form nuclear clusters in mammalian cells (Figures S3A and S3B). The results of 3D structured illumination microscopy (SIM) imaging showed that endogenous CTCF formed small nuclear clusters in live cells (Figures 3A and S3C). The endogenous CTCF protein clusters were also sensitive to 1,6-hexanediol treatment (Figure S3D). Real-time imaging of halo-tagged CTCF revealed a few small CTCF clusters in the early stage of mitotic exit, and these clusters subsequently grew larger as the cell cycle progressed (Figure S3E and Video S6). These results are consistent with previously reported functions of CTCF and chromatin reorganization during mitotic exit (Abramo et al., 2019; Oomen et al., 2019; Zhang et al., 2019). The different reported distribution patterns of CTCF are owing to the various imaging methods applied. As the endogenous CTCF clusters are very small, it is challenging to determine whether other protein factors colocalize with CTCF clusters through imaging techniques. This is why we used the optoDroplet system to enlarge the cluster signals, which are easily observed under low-resolution microscopy.

Video S6. CTCF cluster tracking in HaloTag-CTCF U2OS cells during mitotic exit, related to Figure 3

We sought to obtain functional insights into full-length CTCF in the genome through bioinformatic analyses. The number of CTCF motifs was calculated according to CTCF ChIP-Seq peaks derived from human embryonic stem cells (Ji et al., 2016), as these cells are normal cells cultured *in vitro*, and studies of these cells would reveal the normal functions of CTCF. More than 10% of CTCF-binding sites presented multiple CTCF motifs with regular ChIP and ChIP-exo datasets (Figure 3B and Table S1), which is consistent with the idea that CTCF could execute its functions in chromatin through interactions with motif arrays (Schuijers et al., 2018). CTCF-CTCF loops have been shown to function as insulated neighborhoods (Dowen et al., 2014; Hnisz et al., 2016a; Ji et al., 2016). CTCF insulation scores were calculated by dividing the values of the Cohesin ChIA-PET signals within the loops by the values of the signals of the loops across loop anchors. The loop anchors were then subgrouped into five groups and ranked from high to low based on the insulation scores. The CTCF ChIP-Seq signals of each group were plotted in a violin diagram. The plot indicated that increased insulation scores for loop anchors were associated with stronger CTCF chromatin binding (Figure 3C). The same trend was also observed in the Hi-C data (Figure 3C).

The pIHLIE luciferase reporter is widely used to evaluate the insulation activities of CTCF (Ishihara et al., 2006; Yao et al., 2010). The reporter consists of H19 promoter-driven firefly luciferase and an enhancer with a CTCF insulator between them. pIHLME is a version of pIHLIE with a mutated CTCF insulator sequence. Various numbers of CTCF motifs were inserted into the mutated regions of pIHLME, and a dual luciferase assay was performed. The analyses indicated that increasing the number of CTCF motifs resulted in significant induction of insulator activity (Figure 3D). The CTCF-binding site orientations were the same for every CTCF motif under our design, which may be why the 3× CTCF motifs did not achieve insulation. We further investigated the interactions between CTCF and transcriptional activators in chromatin by performing colocalization analyses with published ChIP-Seq data. The signals of transcriptional activators (OCT4, NANOG, and CBP) and Cohesin ChIP-Seq were plotted at the CTCF-binding peaks. The analyses indicated that the CTCF-binding peaks were associated with a high density of Cohesin binding, as expected, but showed little binding of OCT4, NANOG, and CBP (Figure 3E). These results suggest that CTCF occupies regions with low densities of transcriptional activators in the genome. Note: the detailed biophysical relationships between CTCF and transcriptional activators warrant further investigation in the future.

### Arginine residues in the DBD are frequently mutated in various cancers and are critical for CTCF insulation

**Figure 4:**
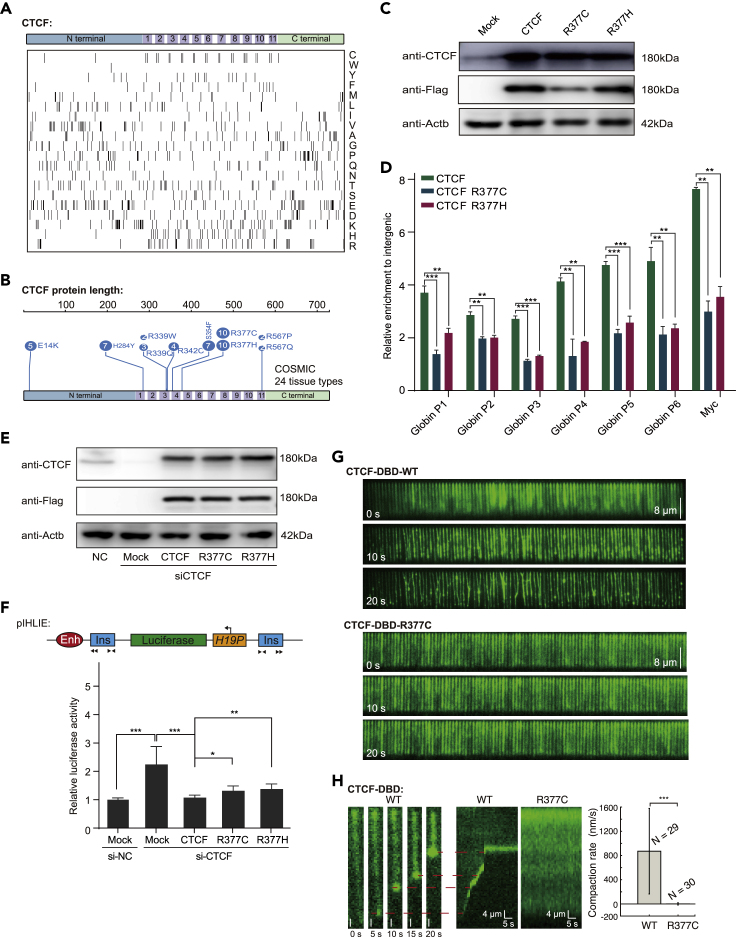
Arginine residues in the CTCF DBD are critical for self-interaction and insulation (A) Amino acid composition of the full-length CTCF protein. Black bars in each row indicate single amino acids, and the single-letter amino acid code is shown in the right panel. (B) Somatic missense mutation hotspot landscape of the CTCF protein. Each number in the circle corresponds to the number of mutations at that amino acid position. The top 7 high-frequency mutation positions were chosen from the COSMIC database. (C) Western blot of total cell lysates of HEK293T cells transfected with eGFP-fused full-length wild-type or cancer-associated mutant CTCF or the EGFP vector (mock). (D) eGFP-fused full-length wild-type or cancer-associated mutant CTCF was transfected into HEK293T cells, and chromatin binding at the C-MYC and b-globin loci was measured by EGFP ChIP–qPCR in transfected cells. Data are presented as the average of three replicates. *p*-values were calculated using two-tailed Student’s *t*-tests (∗∗∗<0.001, ∗∗<0.01). Data are represented as the mean ± SD. The qPCR primer sequences are shown in Table S5. (E) Western blot of total cell lysates of HEK293T cells after the siRNA-mediated knockdown of CTCF and rescue with siRNA resistant 2xFlag-fused full-length wild-type or cancer-associated mutant CTCF or the eGFP vector (mock). (F) Schematic illustration of the pIHLIE reporter used in the luciferase assay (top). Luciferase activities of the pIHLIE construct in the presence of the empty vector (Mock), full-length CTCF, or cancer-derived mutants (R377C and R377H) in HEK293T cells. The luciferase signal was normalized to that of the internal control. Data are presented as the average of three replicates. *p*-values were calculated using two-tailed Student’s *t*-tests (∗∗∗<0.001, ∗∗<0.01, ∗<0.05). Data are represented as the mean ± SD. The black arrow heads represent the direction of CTCF motif. (G) Wide-field TIRFM image showing that the wild-type (top) and R377C (bottom) CTCF DBDs interacted with DNA at the indicated time points. (H) Wide-field TIRFM images showing the compaction of a lambda DNA molecule with 1-μM wild-type CTCF DBD at each specific time point (left). Kymograph showing the compaction of the lambda DNA molecule with the wild-type or R377C CTCF DBD. Compaction rate of the wild-type (*N* = 29) and R377C CTCF DBDs (*N* = 30; right). Data are represented as the mean ± SD. The distributions were statistically compared using the two-tailed Student’s *t*-test (∗∗∗*p* < 0.001).

We next investigated the molecular characterizations of the CTCF DBD via a variety of *in silico* analyses and mutational experiments. First, we noted that the CTCF zinc fingers were preferentially enriched with cysteine, histidine, and arginine residues (Figure 4A). The enrichment with cysteine and histidine residues was expected, as CTCF is a C2H2-type zinc finger transcription factor. The optoDroplet assay revealed that the samples bearing variants with arginine mutations produced significantly fewer protein clusters than those with the wild-type CTCF DBD, so these mutations apparently generate CTCF variants with a reduced ability to form clusters, whereas cysteine and histidine appear to be necessary for the nuclear localization of the CTCF DBD (Figure S4A). Interestingly, the analysis of the COSMIC database using the entire CTCF open reading frame as a query indicated that the CTCF DBD is a cancer mutation hotspot (Figure 4B and Table S2). Enrichment analyses of COSMIC mutations of the CTCF ORF across 24 different tissue types revealed that approximately 20% of CTCF mutations were associated with endometrioid carcinoma.

We generated variants of the two most frequently occurring DBD mutations (R377C and R377H) across 24 different tissue types in the COSMIC database. ChIP–qPCR analyses of the chromatin-binding levels of the aforementioned CTCF variants for cancer mutations indicated significantly reduced binding at both the *C-MYC* and *b-globin* loci (Figures 4C and 4D). The insulation activities of the CTCF mutants were then investigated with previously documented insulator reporters. RNAi knockdown of CTCF decreased the insulator activity of the pIHLIE reporter, and the insulation activity was of a relatively similar magnitude (on average, two-fold) to the previous measurement with Hi-C contact frequency (Chang et al., 2020). The insulator activity could be rescued by the overexpression of a siRNA-resistant version of full-length CTCF. The overexpression of cancer-associated CTCF mutants partially rescued the insulator activities (Figures 4E and 4F). Together, the results indicated that the cancer-associated arginine mutations in the CTCF DBD interfered with the DNA-binding and insulation functions of CTCF.

Previous studies have used the high-throughput single-molecule DNA curtain method (Zhao et al., 2017) to monitor interactions between DNA and heterochromatin protein 1α (HP1α; Larson et al., 2017) and Vernalization 1 (VRN1; Zhou et al., 2019). The authors of these studies speculated that the DNA “shrinking” behavior that they observed occurred owing to a liquid–liquid phase separation mechanism in DNA, suggesting a biological function of gene repression (Larson et al., 2017). We conducted similar experiments in which CTCF DBD was expressed and purified *in vitro*, and its DNA-binding activity was confirmed (Figure S4B). DNA curtain analysis showed that the wild-type protein, but not the R377C mutant variant (from endometrioid carcinoma), could readily bind the DNA curtain, shrink DNA, and form bright fluorescent clusters at the ends of the DNA sequence (Figure 4G and Videos S7a and S7b). Specifically, the compaction rate of the wild-type CTCF DBD was measured as 871 ± 706 nm/s (mean ± s.d., Figure 4H). In comparison, the R377C variant did not shrink DNA under the investigated conditions (Figures 4G and 4H and Video S8) but displayed lower-affinity DNA binding in electrophoretic mobility shift assays (EMSAs; Figure S4C). The differences in DNA binding between the EMSA and DNA curtain experiments were owing to the differences in these technologies. Our results indicate that a potentially pathogenically relevant mutation might compromise the clustering or DNA-binding capacity of CTCF.

Video S7a. CTCF DBD was washed into the flow cell in a high-density DNA region of the DNA curtain setup, related to Figure 4

Video S7b. Example image of CTCF DBD in low-density DNA regions of the DNA curtain setup, related to Figure 4

Video S8. The cancer-associated CTCF DBD mutant R377C was washed into the flow cell of the DNA curtain setup, related to Figure 4

### DNA inhibits CTCF DBD clustering

**Figure 5:**
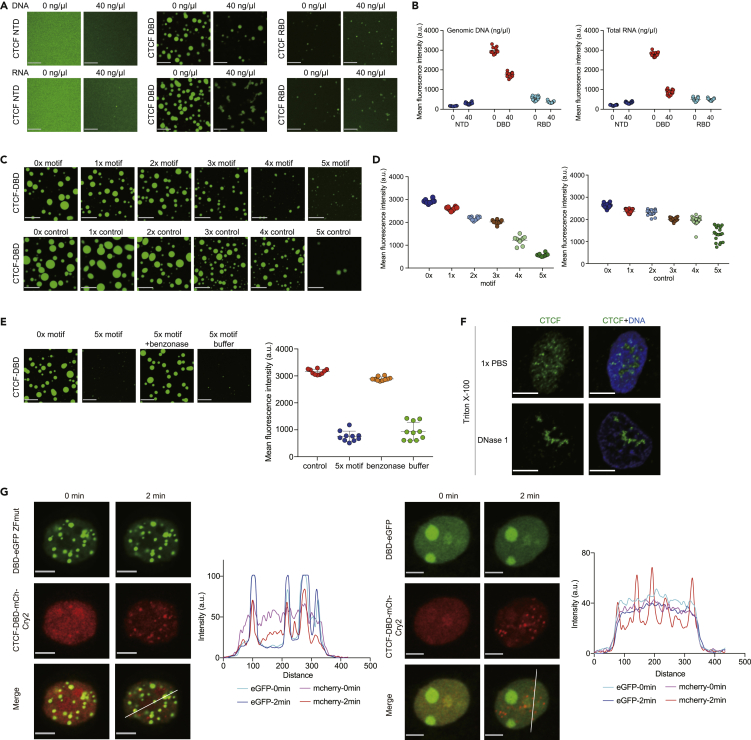
DNA inhibits CTCF DNA-binding domain clustering (A) Representative images of droplet formation resulting from mixing 10 μM CTCF-NTD or CTCF-DBD and CTCF-RBD with 40 ng/μL genomic DNA (top) or 40 ng/μL total RNA (bottom) in the phase buffer. Scale bar, 10 μm. (B) Quantification of the relative fluorescence intensity of droplets under the corresponding conditions. Data are represented as the mean ± SD. (C) Representative images of droplet formation resulting from mixing 10 μM CTCF-DBD with 10 μM CTCF motif dsDNA (top) or 10 μM control dsDNA (bottom) in phase buffer. Scale bar, 10 μm. (D) Quantification of the relative fluorescence intensity of droplets under the corresponding conditions. Data are represented as the mean ± SD. (E) Representative images of droplet formation resulting from mixing 10 μM CTCF-DBD with 10 μM 5× motif dsDNA under treatment with the benzonase or benzonase buffer. Quantification of the relative fluorescence intensity of droplets under the corresponding conditions is shown on the right. Data are represented as the mean ± SD. (F) Immunofluorescence (IF) imaging of CTCF in HEK293T cells before and after the addition of 1× PBS or DNase I. Scale bars, 5 μm. (G) Fluorescence images of a HEK293T cell expressing CTCF-DBD-mCh-Cry2 with DBD-eGFP ZFmut (left) or DBD-eGFP (right) before and after 2 min of stimulation with blue light. Scale bars, 5 μm. The fluorescent intensity profiles at different positions in the optoCTCF-DBD puncta are depicted by the white line before and after stimulation with blue light in the 488- and 561-nm channels.

DNA has been implicated in regulating condensate formation by inducing conformational changes or weak interactions between proteins and DNA (Du and Chen, 2018; Larson et al., 2017; Strom et al., 2017). To investigate the roles of DNA in CTCF DBD clustering, we added genomic DNA to the recombinant purified CTCF NTD, DBD, and RBD for *in vitro* droplet reactions. The results showed that the addition of genomic DNA suppressed CTCF DBD clustering *in vitro*. In contrast, the CTCF RBD formed relatively small clusters compared with the DBD and was insensitive to genomic DNA treatment. As a control, the addition of a similar amount of total RNA showed a similar effect on CTCF DBD clustering but not on CTCF NTD and RBD (Figures 5A and 5B). We then added PAGE-purified CTCF motif-containing DNA oligos to the *in vitro* CTCF DBD droplet system and found that CTCF DBD clustering was eliminated by increasing the number of CTCF motifs, which was rescued by further treatment with benzonase to degrade the DNA but not by treatment with the enzyme working buffer (Figures 5C–5E). We found much weaker effects for the control DNA oligos that did not bind to CTCF (Figures 5C and 5D).

To investigate the relationship between DNA and CTCF clustering in cells, we treated CTCF nuclear clusters with agents known to disrupt bimolecular interactions that are required for condensate formation, such as DNase. DNase treatment led to the formation of CTCF protein clumps from multiple clusters in the nucleus (Figure 5F). Furthermore, CTCF DBD cotransfection with CTCF DBD optoDroplet experiments showed that CTCF DBD localized to the nucleolus, likely owing to the interactions with NPM1 reported previously (Yusufzai et al., 2004), and CTCF DBD did not colocalize with CTCF DBD optoDroplets. In contrast, the CTCF DBD DNA-binding mutants colocalized with the CTCF DBD optoDroplets (Figure 5G). These results further suggested that DNA suppresses the interactions between CTCF DBDs and that our CTCF DBD optoDroplets did not contain DNA. The DNA inhibition of CTCF DBD clustering was consistent with the observation that CTCF does not form large clusters in the nucleus, which may be primed for CTCF homodimerization for Cohesin-mediated loop extrusion.

### More transcription factor DBDs exhibit selective protein–protein interactions

**Figure 6:**
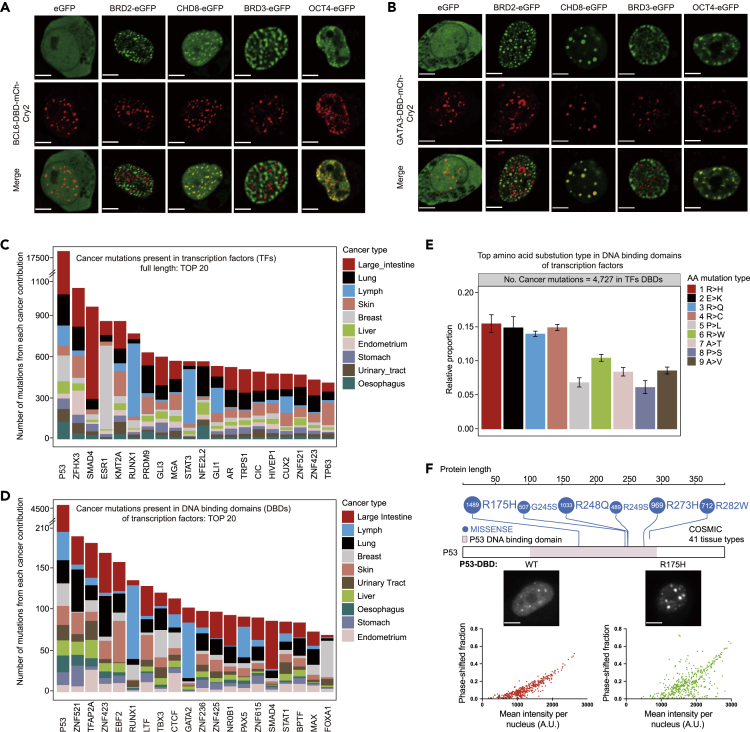
Additional transcription factor DBDs mediate selective protein–protein interactions (A) Representative images of HEK293T cells expressing BCL6-DBD-mCh-Cry2 with eGFP, BRD2-eGFP, CHD8-eGFP, BRD3-eGFP, and OCT4-eGFP. Representative images of blue light-activated cells are shown. Scale bars, 5 μm. (B) Representative images are shown the same as (A) but for GATA3-DBD-mCh-Cry2. Scale bars, 5 μm. (C) Top 20 transcription factors that are frequently mutated in cancers. The types of cancers are listed on the right. (D) Top 20 DBDs of transcription factors that are frequently mutated in cancers. The types of cancers are listed on the right. (E) Most frequent types of missense amino acids in the DBDs of transcription factors in the COSMIC database. (F) Hotspot somatic missense mutational landscape of the P53 protein. The top 6 high-frequency mutation positions were chosen from the COSMIC database (top). Images of the expression of the wild-type P53 (transcription factor) DBD (left bottom) and the P53 R157H cancer mutant DBD (right bottom) fused to mCherry-Cry2 in HEK293T cells. The phase-shifted fraction indicated the area of optoDroplet clusters relative to the total nuclear area in the same cell. Scale bars, 5 μm.

In light of our demonstration that the CTCF DBD clusters formed by self-interaction are capable of mediating the selective interactions of CTCF, we next explored whether this finding may be more generally applicable to other transcription factors. Hence, the optoDroplet assay was performed with two other C2H2-type DBDs (from BCL6 and YY1) and one GATA-type DBD (from GATA3), and the results showed that the DBDs of BCL6, YY1, and GATA3 each formed self-interacting protein clusters in HEK293T cells (Figure S4D). These results suggest that the DBDs of transcription factors may function via extensive protein–protein interactions, in addition to their intrinsic DNA-binding activity. Our findings indicated that the optoDroplet CTCF DBD is capable of interacting with insulator proteins and avoiding high concentrations of transcriptional activators. A similar optoDroplet assay performed with the BCL6 DBD and the GATA3 DBD showed that BCL6-DBD and GATA3-DBD optoDroplets colocalized with CHD8 and OCT4 but not with BRD2 and BRD3 (Figures 6A and 6B). Figure 3A shows that CTCF DBD optoDroplets colocalized with BRD2 and CHD8 but were not coenriched with OCT4 and BRD3. These results suggest that the distribution relationships of BCL6 and GATA3 DBD optoDroplets with insulator proteins and transcriptional activators were different from those of CTCF DBD, implicating that CTCF DBD may have different properties from other DBDs.

Cancer-associated arginine mutations in the CTCF DBD interfered with its DNA-binding and insulation functions, so we considered whether these findings might point toward a potentially widespread mechanism in cancer. By searching the unique gene IDs of transcription factors from the COSMIC database (v87), we identified 174,974 missense variants in 1,254 human transcription factors (Barrera et al., 2016; Deplancke et al., 2016; Lambert et al., 2018), and 501 transcription factors presented 23,313 total missense variants in their DBDs (Figures 6C and 6D and Tables S3 and S4). We noted that the large intestine, lymph nodes, and lungs were the three organs harboring the largest numbers of missense mutations in the DBDs of transcription factors (Figure 6D and Table S4). The average relative contributions of different amino acid substitutions among the mutations found in each cancer type were calculated, and the results showed that arginine was the most frequently mutated amino acid (R > H, R > Q, R > C, and R > W, four of the nine most frequent amino acid mutation types) among the COSMIC mutations in the full-length proteins and DBDs of transcription factors (Figures 6E and S4E). The single most frequently mutated transcription factor, P53, had 7,992 missense mutations in its DBD among 41 different cancers in the COSMIC database (Figure 6F and Table S4). These results suggest that arginine residues are frequently mutated in cancers. We further cloned the wild-type DBD of P53 and a hotspot mutant (R175H) of this DBD into the optoDroplet reporter. Surprisingly, the P53 DBD formed protein clusters, and the cancer-associated P53 DBD mutation obviously affected protein cluster formation in HEK293T cells (Figure 6F). We found that the phase-shifted fraction of the R175H mutation varied considerably compared with the wild-type P53-DBD, suggesting that the R175H mutation of P53-DBD made the clustering unstable. Compared with the CTCF DBD, the results imply that arginine residues contribute differently to protein clustering for different transcription factors. This evidence is consistent with a potential mechanism in which cancer mutations in transcription factor DBDs result in the dysregulation of self-interactions.

## Discussion

The CTCF/Cohesin complex-mediated loop extrusion model explains the molecular basis of 3D genome organization well, but it is still challenging to understand how CTCF/Cohesin chromatin loops block enhancer functions in three-dimensional nuclear organization. Specifically, we demonstrate that the DBD of CTCF undergoes dynamic self-interaction independent of its IDR *in vitro* and in cells with optoDroplet. The CTCF DBD selectively interacts with insulator proteins and avoids transcriptional activators. Other domains of CTCF do not show similar properties. Accordingly, endogenous CTCF forms small protein clusters and binds genomic regions with a high abundance of CTCF motifs but low densities of transcriptional activators, which is consistent with a spatial segregation model of CTCF insulation (Figure S5A). Furthermore, the DBDs of other transcription factors show selective protein–protein interactions, and arginine residues are frequently mutated in various cancers. Our results reveal a previously underappreciated function of the DBD: the ability to engage in selective, dynamic, and transient protein–protein interactions, which provides insights for understanding transcription factor function in development and diseases. These CTCF DBD results inspired us to propose a spatial segregation model in which CTCF selectively interacts with insulator proteins and avoids nuclear positions with a high density of transcriptional activators, which may spatially block the communication of the transcriptional apparatus of enhancers to activate its targeted promoters (Figure S5A). This model was based on the results of CTCF DBD, which warrants further investigation of full-length endogenous CTCF in the future.

Recent studies have shown that CTCF forms small protein clusters in the nucleus, which are required for proper 3D chromatin organization and gene expression (Hansen et al., 2019; Saldana-Meyer et al., 2019). Recombinant purified CTCF and RNA form multimers of more than two megadaltons *in vitro* (Saldana-Meyer et al., 2014). Our current results could be simply explained by the liquid–liquid phase separation (LLPS) concept (Alberti et al., 2019; Brangwynne et al., 2009), but they are not sufficient to reach a conclusion about whether endogenous full-length CTCF forms LLPS because our current evidence is based mainly on studying the CTCF DBD under artificial conditions. Recent quantification results showed that the endogenous concentration of CTCF in mammalian cells is approximately 0.1 μM (Cattoglio et al., 2019), whereas our *in vitro* droplet assay indicated that at a concentration of 10 μM, the CTCF DBD formed phase-separated condensates. We also found that the total CTCF protein levels could not be dramatically induced by transfection of exogenously expressed CTCF, suggesting a possible autoregulation mechanism of CTCF protein, as previously observed (Holzmann et al., 2019; Kung et al., 2015). However, we cannot exclude the possibility that a high concentration of CTCF might result in phase separation of the protein under unusual circumstances, such as senescence in specific cell types (Zirkel et al., 2018) or the concentration of the protein at centrosomes during mitosis (Burke et al., 2005; Xiao et al., 2015). We also believe that our CTCF DBD-mediated protein clusters may play a role in the regulation of small clusters of endogenous CTCF at endogenous concentrations.

The cooperation among transcription factors is usually explained by transcription factor–transcription factor interactions, transcription factor-mediated DNA bending, or combinatorial interactions with the transcriptional machinery (Lambert et al., 2018; Levine et al., 2014; Martinez and Rao, 2012; Spitz and Furlong, 2012); however, the molecular basis of these interactions remains elusive. Here, we serendipitously found that the CTCF DBD undergoes self-interaction and mediates the formation of protein clusters that incorporate insulator proteins but avoid transcriptional activators. The biophysical features of the CTCF DBD that we observed in this study are well correlated with the current knowledge of CTCF insulation, whereas other domains of CTCF do not show these features. The DBD clustering phenomenon is also likely to apply to many other transcription factors, which would provide assays for visualizing the relationships between different transcription factors. The functions of full-length CTCF in cells are also variable; CTCF occupies promoter-proximal regions for gene activation (Kubo et al., 2021; Schuijers et al., 2018) and binds to the distal insulator region, which blocks enhancers from activating gene promoters (Bartolomei, 2009; Hou et al., 2008). In addition, CTCF participates in diverse functions, including DNA replication, DNA repair, Pol II transcription, and splicing (Chernukhin et al., 2007; Hwang et al., 2019; Lang et al., 2017; Shukla et al., 2011; Zhang et al., 2019; Zhao and Dean, 2004). We found different protein‒protein interaction properties for the CTCF DBD and CTCF RBD, indicating that the biophysical properties of the different domains are variable. It is possible that different domains carry out different functions under different environmental conditions. This is also consistent with the diverse functions of CTCF in the nucleus.

The DNA oligo containing the CTCF motif inhibits the CTCF-DBD cluster in an *in vitro* droplet assay. The optoDroplet experiments showed colocalization between CTCF-DBD and its interacting partners, suggesting that CTCF-DBD facilitates protein‒protein interactions. These results reflect the possibility that once CTCF is no longer bound to DNA, it may cluster through the DBD and enrich insulator proteins nearby, which would increase the local concentration of insulator proteins at CTCF-binding sites. CTCF-binding sites usually contain CTCF-binding motif arrays. Interaction with DNA suppresses the DBD-mediated clustering. This setting would facilitate the formation of CTCF dimers for chromatin organization during loop exclusion. This possibility warrants further investigation in the future.

DNA with CTCF-binding motifs inhibited CTCF DBD droplets *in vitro* (Figure 5A), whereas DNA elements with CTCF-binding motifs increased the CTCF insulation activities with the luciferase reporter (Figure 3D). *In vitro* droplet analyses were carried out on purified CTCF DBD proteins, and the results indicated that DNA might inhibit CTCF DBD-mediated protein‒protein interactions. Luciferase assays were performed in cells with full-length CTCF, and the DNA inhibited CTCF DBD clustering, but CTCF formed small protein clusters through its RBDs in cells. On the other hand, the local concentration of CTCF could be further increased with CTCF motif arrays to execute the insulation function. This may be a plausible explanation for why CTCF-binding motifs increased CTCF insulation activities with the luciferase reporter. DNA oligos containing CTCF motifs dissolved CTCF DBD droplets *in vitro*, whereas the lambda DNA was compacted by CTCF DBD, as shown by the analysis with a single-molecule DNA curtain. A possible reason is that the quantity of CTCF DBD proteins was excessive in the DNA curtain experiment, whereas the quantity of DNA molecules was excessive in the *in vitro* droplet assay.

We are just beginning to identify and investigate the activity of CTCF in avoiding transcriptional activators in cells, and standards for defining this avoidance activity have not yet been developed (McSwiggen et al., 2019). The observation that CTCF DBD avoids regions with high densities of transcriptional activators raises more questions than it answers. For example, how does CTCF DBD avoid transcriptional activators in the nucleus? There are approximately 1,200 transcription factors in humans (Lambert et al., 2018). How many of these transcription factors form protein clusters, and do they incorporate relevant factors and avoid opposing factors in the three-dimensional nucleus? Importantly, the disruption of CTCF clustering has been shown to disrupt chromatin looping and dysregulate global gene expression (Hansen et al., 2019). We believe that DNA-binding activity is essential for CTCF functions, but we would like to highlight that the protein–protein interactions mediated by the DBD may play an additional, important role. How the DBD mediates protein interactions and DNA binding in endogenous full-length CTCF proteins is still unknown. It could be that CTCF DBD interacts with other CTCF molecules while bound to DNA, DNA-bound CTCF interacts with other CTCF DBDs through a different domain, or CTCF interacts with other CTCF molecules when it is not bound to DNA. We believe the relationship with DNA binding is worth investigating in the future.

### Limitations of the study

This study revealed an unexpected clustering effect of CTCF DBDs, and further showed the selective protein‒protein interactions for CTCF DBD. However, whether and how the full-length CTCF exhibits similar properties to its DBD has not been documented in the current study. In addition, the functional relationship between CTCF DBD clustering and DNA-binding capacity is also unclear. Therefore, further investigation to address these two limitations would provide further insights into the insulator functions of CTCF in mammalian cells.

## STAR★Methods

### Key resources table

**Table:** 

REAGENT or RESOURCE	SOURCE	IDENTIFIER
**Antibodies**		

Rabbit polyclonal anti-CTCF	Millipore	Cat# 07-729; RRID: AB_441965
Rabbit polyclonal anti-CTCF	Abcam	Cat# ab128873; RRID: AB_11144295
Rabbit polyclonal anti-CTCF	Active motif	Cat# 61311; RRID: AB_2614975
Rabbit polyclonal anti-HaloTag	Promega	Cat# G9281; RRID: AB_713650
Rabbit monoclonal anti-FLAG	Sigma-Aldrich	Cat# F1804; RRID: AB_262044
Rabbit monoclonal anti-BRD2	Cell Signaling Technology	Cat# 5848; RRID: AB_10835146
Mouse monoclonal anti-GAPDH	Proteintech	Cat# 60004-1-Ig; RRID: AB_2107436
Mouse monoclonal anti-Actin	Proteintech	Cat# 66009-1-Ig; RRID: AB_2687938
Donkey anti-Rabbit IgG, Alexa Fluor 488	Invitrogen	Cat# A-21206; RRID: AB_2535792
Donkey anti-Rabbit IgG, Alexa Fluor 568	Invitrogen	Cat# A-10042; RRID: AB_2534017
Rabbit IgG HRP Linked Whole Ab	Sigma-Aldrich	Cat# GENA934-1ML; RRID: AB_2722659
Mouse IgG HRP Linked Whole Ab	Sigma-Aldrich	Cat# GENXA931-1ML; RRID: AB_772209

**Bacterial and virus strains**		

TransT1 Chemically Competent Cell	Transgene	Cat# CD501-02
Transetta (DE3) Chemically Competent Cell	Transgene	Cat# CD801-02

**Chemicals, peptides, and recombinant proteins**		

Halotag-JF549 ligand	Promega	Cat# GA1110
Indole-3-acetic acid	Sigma-Aldrich	Cat# I5148
GSK3b inhibitor CHIR99021	Selleck Chemicals	Cat# S2924
MEK inhibitor PD0325901	Selleck Chemicals	Cat# S1036
YOYO-1	Thermo Fisher	Cat# Y3601
SYBR Safe	Thermo Fisher	Cat# S33102
mLIF	Millipore	Cat# ESG1107

**Critical commercial assays**		

Lipofectamine 2000	Mei5bio	Cat# MF135-1
Lipofectamine RNAiMAX	Thermo Fisher	Cat# 13778075

**Deposited data**		

Microscopy images and blots scans data	This paper	Mendeley data: https://doi.org/10.17632/ssj66hy8sd.1

**Experimental models: Cell lines**		

HEK293T cells	ATCC	CRL-3216
U2OS cells	ATCC	HTB-96
V6.5 murine embryonic stem cells	Richard A. Young laboratory	N/A
mESC CTCF-GFP-mAID	Wei Xie laboratory	N/A

**Oligonucleotides**		

Guide RNA sequences (sgRNAs) for Chr3q29 loci: TGATATCACAG	This Paper	N/A
Primers for plasmid constructs, see Table S5	This Paper	N/A
Primers for qPCR, see Table S5	This Paper	N/A
EMSA C-MYC probes: CTGCTGCCAGTAGAGGGCACACTTA	This Paper	N/A
EMSA NC probes: TCTCCTATGACTCGTCCAT	This Paper	N/A
Sence-CTCF-siRNA: GCGCUCUAAGAAAGAAGAUUCCUCU	This Paper	N/A
Antisence-CTCF-siRNA: AGAGGAAUCUUCUUUCUUAGAGCGC	This Paper	N/A
Sence-Ctrl-siRNA: CGUACGCGGAAUACUUCGATT	This Paper	N/A
Antisence-Ctrl-siRNA: UCGAAGUAUUCCGCGUACGTT	This Paper	N/A

**Recombinant DNA**		

pHR-mCherry-CRY2	Shin et al. (2017)	Addgene Plasmid #101221
pHR-FUSN-mCherry-CRY2	Shin et al. (2017)	Addgene Plasmid #101223
pHR-CTCF(DBD)-mCherry-CRY2	This Paper	N/A
pHR-CTCF(RBD)-mCherry-CRY2	This Paper	N/A
pHR-CTCF(NTD)-mCherry-CRY2	This Paper	N/A
pHR-CTCF(R-I)-mCherry-CRY2	This Paper	N/A
pHR-CTCF(K-I)-mCherry-CRY2	This Paper	N/A
pHR-CTCF(H-I)-mCherry-CRY2	This Paper	N/A
pHR-BCL6(DBD)-mCherry-CRY2	This Paper	N/A
pHR-GATA3(DBD)-mCherry-CRY2	This Paper	N/A
pHR-P53(DBD)-mCherry-CRY2	This Paper	N/A
pHR-YY1(DBD)-mCherry-CRY2	This Paper	N/A
pET28a-SUMO-eGFP	This Paper	N/A
pET28a-SUMO-CTCF(DBD)-eGFP	This Paper	N/A
pET28a-SUMO-CTCF(NTD)-eGFP	This Paper	N/A
pET28a-SUMO-CTCF(RBD)-eGFP	This Paper	N/A
pET28a-SUMO-meGFP	This Paper	N/A
pET28a-CTCF(DBD)-meGFP	This Paper	N/A
pET28a-SUMO-CTCF(DBD)-meGFP	This Paper	N/A
pX332-eGFP	This Paper	N/A
pX332-BRD2-eGFP	This Paper	N/A
pX332-CHD8-eGFP	This Paper	N/A
pX332-BRD3-eGFP	This Paper	N/A
pX332-OCT4-eGFP	This Paper	N/A
pX332-NANOG-eGFP	This Paper	N/A
pX332-SOX2-eGFP	This Paper	N/A
pX332-Halo-eGFP	This Paper	N/A
pIHLME	Yao et al. (2010)	N/A
pIHLIE	Yao et al. (2010)	N/A
phRG-TK	Yuanchao Xue laboratory	N/A
pcDNA-CTCF-2xFLAG	This Paper	N/A
pcDNA-CTCF(R377C)-2xFLAG	This Paper	N/A
pcDNA-CTCF(R377H)-2xFLAG	This Paper	N/A
pLH-sgRNA-Chr3	This Paper	N/A
pHAGE-TO-dCas9-3xmcherry	Ma et al., 2015	Addgene Plasmid #64108
pHAGE-TO-dCas9-3xmcherry-CTCF	This Paper	N/A
pHAGE-TO-dCas9-3xmcherry-CTCF(dDBD)	This Paper	N/A

**Software and algorithms**		

ImageJ	National Institutes of Health (NIH)	https://imagej.nih.gov/ij/
Volocity (v.6.3)	PerkinElmer	https://www.perkinelmer.com/
FrapBot	(Kohze et al., 2017)	http://frapbot.kohze.com/
GraphPad (v.7)	Prism	https://www.graphpad.com/scientific-software/prism
Bowtie2 (v2.3.5.1)	Langmead and Salzberg. (2012)	http://bowtie-bio.sourceforge.net/bowtie2/index.shtml
MACS2 (v2.2.5)	Zhang et al. (2008)	https://github.com/taoliu/MACS
deeptools (v3.4.3)	Ramirez et al. (2016)	https://deeptools.readthedocs.io/en/develop/
Homer (v4.10)	Heinz et al. (2010)	http://homer.ucsd.edu/homer/motif/
BEDTools (v2.27.1)	Quinlan and Hall. (2010)	https://github.com/arq5x/bedtools2

**Other**		

PerkinElmer UltraView VoX spinning disk confocal microscopy	Nikon	N/A
Nikon A1RSi confocal microscopy	Nikon	N/A
DeltaVision OMX System	GE Healthcare	N/A
JASPAR	Khan et al. (2018)	https://jaspar.genereg.net/
COSMIC	Tate et al. (2019)	https://cancer.sanger.ac.uk/cosmic
hESC CTCF ChIP-seq	GSE69646	Ji et al. (2016)
mESC CTCF ChIP-exo	GSE98671	Nora et al. (2017)
mESC ChIP-seq datasets (SMC1, OCT4, NANOG, and CBP)	GSE44286	Whyte et al. (2013)

### Resource availability

#### Lead contact

Further information and requests for resources and reagents should be directed to and will be fulfilled by the lead contact, Xiong Ji (xiongji@pku.edu.cn).

#### Materials availability

Cell lines and plasmids generated in this study will be shared by the lead contact upon reasonable request.

### Experimental model and subject details

#### Cell culture

HEK293T cells (ATCC, CRL-3216) and U2OS cells (ATCC, HTB-96) were cultured in 10% fetal bovine serum (FBS, Gibco, 10099–141) in Dulbecco’s modified Eagle’s medium (DMEM, Gibco, 11995–065) supplemented with 1% penicillin and streptomycin (Gibco, 15140–122) at 37°C with 5% CO2 in a humidified incubator. Mouse embryonic stem cells (mESCs), a gift from Dr. Richard A. Young (Whitehead Institute, USA), were grown on 0.2% gelatinized (Sigma, G1890) plates in 2i medium consisting of ES-DMEM (Millipore, SLM-220-M), 15% FBS (Gibco, 10099–141), an extra 0.5 mM L-glutamine (Gibco, 25030–081), 0.1 mM b-mercaptoethanol (Millipore, ES-007-E), 1% penicillin‒streptomycin (Gibco, 15140–122), 0.5× nonessential amino acids (Millipore, TMS-001-C), 1000 U/mL LIF (Millipore, ESG1107), 1 μM PD0325901 (Selleck, S1036), and 3 μM CHIR99021 (Selleck, S1263). For imaging experiments, cells were grown on glass-bottom dishes (Cellvis D35C4-20-1.5-N) or 35 mm glass-bottom dishes (MatTek, USA).

#### Cell treatments

For the 1,6-hexanediol treatment assay, HEK293T cells were grown on 35 mm glass-bottom dishes (MatTek, USA) in 1 mL of culture medium. Before 1,6-hexanediol treatment, a repetitive activation cycle was applied for cluster formation (the activation program was documented in the optoDroplet assay section). We used different concentrations of 1,6-hexanediol to examine the dynamics of protein clusters, and the sensitivities of our analyses (live-cell imaging, CTCF-DBD droplets, IF) were variable. For the more stable clusters or less sensitive detection methods, such as live-cell imaging analyses, we used a concentration of 10% 1,6-hexanediol. For more dynamic clusters or more sensitive analyses, such as CTCF-DBD optoDroplet analyses and *in vitro* droplet assays, we used a concentration of 3% 1,6-hexanediol. DNase I digestion for immunofluorescence: HEK293T cells were grown on coverslips, permeabilized with 0.1% Triton X-100 (Sigma Aldrich, T8787) in 1× PBS for 2 min, washed immediately with 1× PBS, and immediately treated with DNase I (0.5 U/μL, Thermo, EN0523) in 1× DNase dilution buffer at 37°C for 10 min. Then, the cells were fixed with 4% paraformaldehyde for 10 min and processed for immunofluorescence.

### Method details

#### Molecular cloning

For optoDroplet plasmids, human CTCF NTD (residues 1–265), zinc finger domain (residues 266–577), CTD (residues 578–727), DBD (residues 322–460), IDR (residues 558–677), and RBD (residues 520–727), BCL6 DBD (residues 518–618), GATA3 DBD (residues 263–313), YY1 DBD (residues 325–407) and P53 DBD (residues 102–292) were subcloned into pHR-FUSN-mCh-Cry2 to replace the coding region of FUSN using Hi-Fi NEBuilder (NEB, E2621S). The pHR-mCh-Cry2 (negative control) and pHR-FUSN-mCh-Cry2 plasmids were obtained from Brangwynne’s laboratory.

For the optoDroplet protein‒protein interaction assay, human BRD2, BRD3, NANOG, CHD8 (residues 2240–2582), mouse OCT4 and SOX2 were subcloned into the px332 plasmid with a substitute for the Cas9 coding region (gift from Dr. Jiazhi Hu, Peking University, China) and fused with an eGFP sequence in the C-terminus. For protein purification plasmids, human CTCF DBD fused with eGFP in the C-terminal with a 15-amino acid linker sequence (EFGAPGSAGSAAGSG) was subcloned into the pET28a-SUMO plasmid (a gift from Dr. Yanli Wang, CAS, China) at multiple clone sites using Hi-Fi NEBuilder (NEB, E2621S). The plasmid with the 6×His-SUMO tag removed was constructed by inserting the HRV3C recognition sequence after the 6×His-SUMO sequence in the pET28a-SUMO vector.

For luciferase reporter plasmids, the N×Tandem CTCF binding sequence (TGCCAGTAGAGGGCACAC, n = 0, 3, 6, 9, 12, 21) was synthesized by Ruibiotech Co., Ltd. (Beijing, China) and cloned into the Pst I site of the pIHLME plasmid. Full-length and cancer mutant (R377C, R377H) CTCF with a 2×Flag tag at the C-terminus were subjected to silencing mutations at siRNA sites by PCR-directed mutagenesis with the PCR-directed mutagenesis primers listed in Table S5 and then subcloned into the pcDNA plasmid at the RBS site using Hi-Fi NEBuilder (NEB, E2621S). pIHLIE and pIHLME were gifts from Dr. Mitsuyoshi Nakao (Kumamoto University, Japan) (Ishihara et al., 2006) and Dr. Hongjie Yao (CAS, China), and phRG-TK was a gift from Dr. Yuanchao Xue (CAS, China).

For dCas9 targeting experimental plasmids, full-length CTCF and DBD-truncated CTCF were cloned into the XhoI restriction site of the pHAGE-TO-dCas9 plasmid.

To generate an endogenous HaloTag-CTCF cell line, coding sequences of Cas9 from *S*. *pyogenes* were inserted into pHAGE-TO-DEST, resulting in pHAGE-TO-Cas9. The guide RNA targeting the CTCF gene spanning the start codon ATG was designed and subcloned into the expression vector pLH-sgRNA1, resulting in pLH-sgRNA1-CTCF. The donor plasmid for knock-in of HaloTag into CTCF consists of the 809 bp left arm upstream of the start codon, HaloTag and the 756 bp right arm downstream of the start codon. The left arm and right arm of the HaloTag-CTCF donor were amplified from U2OS genomic DNA by PCR. The Golden Gate cloning method was used to assemble the HaloTag-CTCF donor into pDONOR to generate pDONOR-HaloTag-CTCF. The plasmids were sequenced to confirm that they were correct.

#### HaloTag-CTCF stable cell generation

Human U2OS cells were cultured on 35 mm dishes to reach 30–50% confluency at transfection. Two hundred nanograms of pHAGE-TO-Cas9, 600 ng of pLH-sgRNA1-CTCF, and 600 ng of pDONOR-HaloTag-CTCF were cotransfected using Lipofectamine 2000 reagent. Then, the culture medium was replaced by fresh medium with 2 nM HaloTag-JF-549 after 6 h. The cells were incubated for another 24–48 h before examining the knock-in efficiency. Fluorescent imaging was used to check the proper localization of HaloTag-CTCF, and flow cytometry was used to select the positive cells. Successful HaloTag-CTCF knock-in U2OS cells were selected using BD FACS Aria III equipped with 561 nm excitation lasers. The emission signals were detected using a filter at 610/20 nm (wavelength/bandwidth) for HaloTag-JF549. Positive cells were pooled into chilled DMEM containing 20% fetal bovine serum and 1% penicillin and streptomycin. The localization of HaloTag-CTCF was examined again under the microscope after two weeks. The resulting heterozygous cell was named U2OS HaloTag-CTCF.

#### Immunofluorescence

Cells were plated on glass coverslips in 6-well plates, washed with prewarmed 1×PBS three times, and fixed in 4% paraformaldehyde (VWR BT140770) in PBS for 10 min at room temperature (RT). After washing, the cells were permeabilized in 0.2% Triton X-100 (Sigma Aldrich, T8787), 1% BSA (Sigma, V900933) for 15 min at RT. After a wash with 1×PBS, the cells were incubated with 2% BSA at RT for at least 40 min and subsequently incubated with primary antibodies (anti-CTCF, Millipore 07–729/Active motif 61311/Abcam ab128873, 1:800 dilution) in 1% BSA overnight at 4°C. After three washes with 1×PBS, 1% BSA, 0.1% Tween 20 for 10 min, the cells were incubated with Alexa Fluor-tagged secondary antibody (donkey anti-rabbit IgG, Alexa Fluor 488, Invitrogen, A-21206, at a 1:1000 dilution) in the dark for 1 h at room temperature. Then, the cells were washed three times, mounted with a fluorescent mounting solution with DAPI (ZSGB-BIO, ZLI-9557) and Vectashield (Vector Laboratories, H-1000), sealed with colorless nail polish, and imaged on a NIKON A1RSi + confocal microscope with a 100×/1.45 oil objective using NIS-Elements software (Nikon, USA).

#### Focus calling and statistical analysis

HEK293T cells were imaged for cluster quantification at the maximal projection of the z-stack. We collected HEK293T cells with a similar diameter (∼10 to 20 μm) and called clusters by using the “Object Counter3D” plugin in FIJI (https://imagej.nih.gov/ij/plugins/track/objects.html). For each group, the “Threshold” parameter was determined to ensure that the clusters adjacent to each other could be recognized as individual objects. In detail, CTCF clusters were identified by setting the minimal size filter to “10” voxels and the lowest threshold of intensity to at least “92” for the untreated group and “64” for the 1,6-hexanediol treatment group. Cluster numbers of 20 individual cells were collected for each group and compared using an unpaired two-tailed Student’s t test. The plots were generated using GraphPad Prism 7.

#### Fluorescence microscopy and live-cell imaging

U2OS HaloTag-CTCF cell imaging was carried out on a DeltaVision OMXTM V4 imaging system (GE Healthcare, USA) equipped with a 63×/1.42 Plan Apo oil-immersion objective (Olympus, Japan), equal to a pixel size of 80 nm in the images. U2OS HaloTag-CTCF cells were cultured on No. 1.0 glass-bottom dishes (MatTek, USA). The microscope stage incubation chamber was maintained at 37°C and 5% CO2. HaloTag-JF549 was excited at 561 nm, and its emission was collected using a filter at 609/37 nm (wavelength/bandwidth). Imaging data were acquired by DeltaVision Elite imaging (GE Healthcare, USA) software. HaloTag-CTCF cluster formation was tracked for 4 h, and images were collected every 15 min for 240 min. To minimize photobleaching and phototoxicity, only one focal plane in each sample was used for tracking. We determined mitotic exit as follows: the metaphase was identified under the microscope, and the metaphase after 2.5 h was defined as the mitotic exit stage by following the protocol published previously (Albini et al., 2015; Grimm et al., 2015; Naumova et al., 2013). The image size was adjusted to show two daughter cells, and intensity thresholds were set based on the ratios between nuclear focal signals and nuclear background fluorescence. Images for the tracking of CTCF cluster formation in each sample were scaled to the same minimal and maximal fluorescence. For the representative images, the raw data were deconvoluted by softWoRx software (GE Healthcare, USA) using the enhanced ratio method. The 3D SIM images of U2OS HaloTag-CTCF cells were obtained on a Nikon N-SIM imaging system. The images were further processed by Fiji software (https://fiji.sc/).

#### OptoDroplet assay

For the optoDroplet assay, HEK293T cells were plated at ∼40% confluency in a 35 mm glass-bottom dish (Cellvis D35C4-20-1.5-N) one day before transfection. OptoDroplet plasmids were transfected into HEK293T cells for approximately 24 h. Then, the cells were light-activated and photographed, as documented (Shin et al., 2017). Briefly, the repetitive activation cycle was applied by varying activation intervals, i.e., the 488 nm activation duration was fixed to 1 s, light power was 4.5%, sensitivity was 111, the 561 nm imaging duration was fixed to 200 ms, light power was 10.5%, and sensitivity was 127 in all measurements. Image capture and analyses were performed on a PerkinElmer UltraView VoX spinning disk microscope. For image analysis, the phase-shift fraction and the mean fluorescence intensity of the nucleus were measured by Volocity software (PerkinElmer) from.tiff files. Statistical analyses were carried out with GraphPad Prism 7. We used the purified mCherry protein as an indicator to estimate the concentration of opto-CTCF-DBD proteins. Based on the BCA assay (23225, Thermo Scientific), the purified mCherry was approximately 20 mg/mL. A serial dilution of mCherry was made to generate a standard curve using western blotting. HEK293T cells were transfected with opto-CTCF-DBD plasmids using Lipofectamine 2000 (MF135-1, Mei5bio). After 24 h, 400,000 mCherry-positive cells were collected using FACS (Aria III, BD Biosciences) and dissolved in 40 μL of protein loading buffer. Eight microliters of protein sample was blotted using the anti-mCherry antibody (26765-1-AP, Proteintech), and the intensity of the blotting band was measured by ImageJ to estimate the concentration of opto-CTCF-DBD proteins. As approximately 0.352 ng of mCherry could be detected in 80,000 opto-CTCF-DBD cells, the transfected CTCF-DBD concentration was measured to be 182 nM in the nucleus (diameter = 12 μm).

For FRAP experiments, light-activated cells were immediately imaged three times before bleaching at 3 s intervals, as described above. Spots of ∼1.5 μm diameter in light-induced clusters were bleached with a 100% laser power of 561-nm laser for 2 s. After bleaching, recovered cells were imaged every 5 s at 561 nm. Intensity traces were measured using Volocity software (PerkinElmer) at the bleached, background, and unbleached regions with a spot of 2 μm diameter in at least five clusters. The intensity profile was then imported into the FrapBot website (http://frapbot.kohze.com/) (Kohze et al., 2017). The recovery time constant (tau), the half time of recovery (t1/2), and the apparent diffusion constant (D) were automatically calculated by background normalization and standard exponential curve fitting. The mobile fraction (Mf) was calculated by the formula Mf = (Ie-Io)/(Ipre-Io), where Ie is the final recovered intensity, Io is the low-value intensity after bleaching, and Ipre is the prebleach intensity. Graph and statistical analyses were carried out with GraphPad Prism 7.

For the optoDroplet protein‒protein interaction assay. HEK293T cells were cotransfected with eGFP, OCT4-eGFP, and CDH8-eGFP with opto-CTCF DBD. Time-lapse images were captured every 6 s over 3 min at 488 nm and 561 nm, as detailed in the “OptoDroplet assay” section, on a Nikon A1RSi laser scanning confocal microscope equipped with a temperature stage at 37°C. The light intensity of both GFP and mCherry captured at 0 min and 1 min was measured along with the line that passed CTCF DBD clusters. The results were plotted with GraphPad Prism 7 with at least five lines for each cell.

For relative position analysis of the opto fusions with eGFP fusions, HEK293T cells were globally activated using a 488-nm laser for up to 3 min to form clusters, and images were instantly captured at 488 nm and 561 nm. The images were analyzed by Volocity software.

#### Protein purification for the *in vitro* droplet assay

The protein purification plasmids were transformed into the *E*. *coli* Transetta (DE3) strain (Transgene). A fresh bacterial colony was inoculated into 20 mL of LB medium containing 50 μg/mL kanamycin (AMRESCO, 0408) and grown overnight at 37°C. The overnight culture was diluted 1:15 in 300 mL of LB with fresh kanamycin and grown for another 2 h at 37°C. After cooling to 18°C, 0.5 mM IPTG was added to the culture. Cells were harvested after 20 h of further growth, washed twice with 1×PBS, and stored at −80°C. Cells containing EGFP alone were treated similarly, except that they were grown at 22°C after adding IPTG. Pellets from 600 mL of cells were resuspended in 20 mL of Buffer A (20 mM Tris-HCl pH = 8.0, 300 mM NaCl, 10 mM imidazole, and protease inhibitors (Roche, 11873580001, 1:50×) and sonicated (99 cycles of 5 s ON, 9 s OFF). Lysates were mixed with polyethyleneimine (Sigma, P3143) pH = 7.0 to a final concentration of 0.3%–0.4% (w/v) before centrifugation at 13,000 × g for 30 min at 4°C. The supernatant with the addition of 20 μM ZnCl_2_ was loaded onto a 3 mL Ni-NTA agarose column (QIAGEN No. 160028558) prebalanced with Buffer A (20 mM Tris-HCl pH = 8.0, 300 mM NaCl, 10 mM imidazole), followed by a washing process using approximately 200 mL of Buffer B (20 mM Tris-HCl pH = 8.0, 300 mM NaCl, 20 mM imidazole). After that, the protein was eluted with 4 × 2 mL Buffer C (20 mM Tris-HCl pH = 8.0, 500 mM NaCl, 300 mM imidazole). SDS‒PAGE was used to qualitatively evaluate the purity of the eluted protein. High-quality sections were combined and dialyzed against Buffer D (20 mM Tris-HCl pH 8.0, 500 mM NaCl) and then concentrated using Amicon Ultra centrifugal filters (Millipore, 30K MWCO) for further use.

#### Protein purification for DNA curtain assay

For the DNA curtain assay, sequences encoding wild-type and cancer mutant R377C CTCF DBD were cloned into a pET21a vector with an Intein-CBD tag at the C-terminus. The plasmids were transformed into the BL21 strain supplemented with 0.1 mM ZnCl_2_. Protein expression was induced at an OD 600 of 0.6 with 0.5 mM IPTG, and the cells were grown overnight at 16°C. Bacteria were pelleted and resuspended in lysis buffer (20 mM Tris-HCl, pH = 8.0, 500 mM NaCl, 0.1% Triton X-100, 1% (v/v) glycerol, 0.1 mM ZnCl_2_, 1 mM DTT, and 1 mM PMSF) and then sonicated for 10 min on ice. The total lysate was treated with 0.2% (w/v) polyethyleneimine and clarified by centrifugation at 18,000 × g for 30 min at 4°C. The supernatant was loaded onto 5 mL of chitin resin (NEB, S6651) prebalanced with column buffer (20 mM Tris-HCl, pH = 8.0, 500 mM NaCl, and 0.1 mM ZnCl_2_). The column was washed with 50 mL of column buffer and eluted by overnight incubation with column buffer containing 50 mM DTT. The fractions containing eluted protein were combined, and 100 mM DTT was added and incubated for 30 min at 60°C. After brief cooling on ice, the protein was dialyzed against two buffer changes of 1 L storage buffer (20 mM Tris-HCl, pH = 8.0, 500 mM NaCl, 0.1 mM ZnCl_2_, and 10 mM DTT). Finally, the protein was concentrated and measured by absorbance at 280 nm. An electrophoretic mobility shift assay (EMSA) was used to confirm the binding activity of CTCF DBD or CTCF DBD R377C.

#### Protein concentration measurements

The protein concentration was determined by entering the amino acid sequence into the Protein Concentration Calculator web server (https://www.aatbio.com/tools/calculate-protein-concentration) to calculate the molecular weight and extinction coefficient. We measured the protein absorbance at 280 nm using a spectrophotometer and divided the absorbance value by the extinction coefficient to obtain the concentration of the purified protein in the solution.

#### DNA oligonucleotide preparation

Single-stranded DNA oligonucleotides were synthesized by Ruibiotech Co., Ltd. (Beijing, China). Double-stranded DNA oligonucleotides were generated by annealing sense and antisense ssDNA oligos in annealing buffer (20 mM Tris-HCl pH 7.5, 50 mM NaCl) while ramping down from 95°C to 25 °C at a rate of 1°C/min.

#### *In vitro* droplet assay

Recombinant protein and double-stranded DNA oligonucleotides were added to solutions at varying concentrations with the indicated final salt concentrations in 20 mM Tris-HCl, pH = 8.0 buffer. Ten microliters of protein solution was incubated at room temperature for 10 min, immediately loaded onto a 96-well or 384-well glass bottom plate (Cellvis, P96-1.5H-N, P384-1.5H-N) and then imaged by a PerkinElmer UltraView VoX spinning disk microscope or Nikon A1 laser scanning confocal microscope with a 63×/1.4 oil objective. Time-lapse imaging was captured with identical microscopy settings every 3 s over 10 min. For image analysis, the mean fluorescence intensity of droplets was measured by ImageJ (NIH) from.tiff files at the center of similar-sized droplets. Statistical analyses were carried out with GraphPad Prism 7.

#### EMSA

C-MYC (5′-CTG CTG CCA GTA GAG GGC ACA CTT A-3′) or NC probes (5′-TCT CCT ATG ACT CGT CCA T -3′) were prepared at a final concentration of 2 μM in annealing buffer (40 mM Tris-HCl pH 8.0, 50 mM NaCl, and 10 mM MgCl_2_). The DNA binding reaction was performed with a 0.2 μM DNA probe in binding buffer (20 mM Tris-HCl pH = 8.0), 150 mM NaCl, 0.1 mM ZnCl_2_, and 1 mM DTT). CTCF DBD protein at serial dilutions of 0, 0.1, 0.2, 0.4, 0.8, 1.6, and 2 μM was mixed well with DNA probes at different concentrations for 30 min at RT. Then, the mixture was resolved by 12% nondenaturing polyacrylamide gel in 0.5× TBE buffer. The DNA was stained with SYBR Safe dye. The obtained image was analyzed with ImageJ.

#### DNA curtains

For the DNA curtain assay, a custom-built prism-type total internal reflection fluorescence (TIRF) microscope with an OBIS 488-nm laser mounted (Nikon Inverted Microscope Eclipse Ti-E) was constructed (Qi and Greene, 2016; Zhao et al., 2017). A 20% laser power was used for all experiments, and the real laser powers before the prism were measured as 9.9 mW. A DNA curtain experiment was set up, the 488-nm laser (20% laser power) was turned on, and data were obtained by acquiring single 100 ms frames at 1 s intervals. The experiment included two steps: (i) Lambda DNA substrates were washed with working buffer (20 mM Tris-HCl pH 8.0, 150 mM NaCl, 0.1 mM ZnCl_2_, 1 mM DTT, 0.2 nM YOYO-1—a green fluorescent dye, and 0.2 mg/mL BSA) for 120 s at room temperature with a flow rate of 0.4 mL/min (ii) Then, 1 μM CTCF DBD or CTCF DBD R377C protein solution was used to wash the flowcell for 90 s at the same flow rate of 0.4 mL/min. When the protein samples reached the flowcell and started to interact with lambda DNA (homer predicted 123 CTCF motifs in lambda DNA), the time was counted as 0. The time points at 10 s and 20 s were plotted. The data analysis of the shrinking behavior in DNA curtains (Figure 4G) was the same as in the previous reference related to VRN1 (Zhou et al., 2019). In the kymographs in Figure 4H, “compaction rate (nm/s)” is the slope of the imaging track of the DNA substrate end, defining how fast the DNA was shrinking.

#### ChIP‒qPCR analyses

HEK293T cells were seeded onto 10 cm plates and transfected with 6 μg of plasmids (vector (mock), wild-type full-length CTCF, or cancer mutants (R377H, R377C) fused with 2×Flag) at 80–90% confluency using Lipofectamine 2000 reagent according to the manufacturer’s instructions. After 24 h, the medium was replaced with fresh medium. Twenty-four hours later, the cells were suspended in 0.25% trypsin and inactivated with DMEM. The resuspended cells were adjusted to 1 million cells/mL, fixed with 1% (wt/vol) formaldehyde and incubated at room temperature for 10 min. Then, 0.125 mM glycine was added and incubated for 5 min to quench the formaldehyde. The cells were washed twice with cold PBS and pelleted at 2500 rpm for 5 min at 4°C. ChIP‒qPCR was performed following a previously published protocol (Haring et al., 2007). Three million fixed cells were resuspended in 1 mL of sonication buffer (20 mM Tris HCl, pH 8.0, 150 mM NaCl, 2 mM EDTA, 0.1% SDS, 1% Triton X-100, 5 mM CaCl_2_) and sonicated using a Biorupter with the following program: high energy, 30 s ON, 60 s OFF, 20 cycles. Sonicated lysates were cleared twice by centrifugation at 12,000 rpm for 10 min at 4°C. Fifty microliters was reserved for input, and the rest was incubated overnight at 4°C with 30 μL of magnetic beads and 1 μL of anti-Flag antibodies (Sigma, F1804). The beads were pelleted and washed once with sonication buffer (20 mM Tris-HCl pH 8.0, 150 mM NaCl, 2 mM EDTA, 0.1% SDS, 1% Triton X-100), once with high-salt wash buffer (20 mM Tris-HCl pH 8.0, 500 mM NaCl, 2 mM EDTA, 0.1% SDS, 1% Triton X-100), once with LiCl wash buffer (10 mM Tris-HCl pH 8.0, 250 mM LiCl, 1 mM EDTA, 1% NP-40), and three times with TE buffer (1 mM EDTA, 10 mM Tris-HCl pH 8.0), and the beads were eluted with 300 μL of elution buffer (50 mM Tris-HCl pH 8.0, 10 mM EDTA, 1% SDS). Then, 4 μL of 10 mg/mL proteinase K and 2 μL of 5 M CaCl_2_ were added. The mixed sample was incubated for 6–10 h at 65°C and inactivated with protease at 80°C for 20 min. The solution was then incubated with 2 μL of RNase A for 30 min at 37°C. ChIP DNA was reverse cross-linked and purified by a DNA purification kit (Megen, D2111-03). ChIP‒qPCR was performed using 2×RealStar Green Mixture (Vazyme, Q711) on a Bio-Rad CFX Connect™ Real-Time PCR Detection System. qPCR primers were designed based on the ChIP-Seq dataset published previously (Hnisz et al., 2016b; Ibarra et al., 2016); for details, see Table S5.

#### Luciferase assay

HEK293T cells were cultured in 24-well plates at 30∼50% confluency at transfection. Then, 0.1 pmol of N×modified pIHLME and 0.1 pmol of phRG-TK were cotransfected into HEK293T cells using Lipofectamine 2000 reagent. The cells were cultured for 24 h and lysed, and luciferase activity was measured by a BioTek Cytation5 chemiluminescence detector using a Dual-Luciferase assay kit (Promega, E1910). The relative luciferase activity was calibrated by Renilla luciferase activity and compared with each other using Student’s t test.

For insulator activity analyses of different CTCF mutants, 80,000 trypsinized HEK293T cells were transfected with 100 nM CTCF or control siRNA oligonucleotides (synthesized by Ruibiotech Co., Ltd. (Beijing, China), listed in Table S5) using Lipofectamine RNAiMAX reagent (Invitrogen, 13778150) in 24-well plates. After 24 hr of culture, 50 nM oligonucleotides, 0.1 pmol of CTCF series plasmids, 0.02 pmol of pIHLIE, and 0.02 pmol of phRG-TK were cotransfected into HEK293T cells using Lipofectamine 2000 reagent. A blank vector was used as a mock control. Luciferase activities were measured after plasmid transfection for approximately 30 hr. The relative luciferase activity was calibrated by Renilla luciferase activity and compared with each other using Student’s t test. All luciferase assays were performed in triplicate and repeated at least three times.

#### SgRNA-dCas9 target assay

U2OS cells were plated at ∼40% confluency in a 35 mm glass-bottom dish one day before transfection. Two hundred nanograms of sgRNA-Chr3 and 2 μg of dCas9-mch-CTCF and DBD-deleted mutants were transfected into U2OS cells for approximately 24 h, and then BRD2 immunofluorescence was performed as described in the Immunofluorescence assay section. Finally, the enrichment of the fluorescence intensity of the BRD2 signal within 0.9 μm^2^ of the center of the targeted foci related to the nuclear mean fluorescence intensity was calculated.

### Quantification and statistical analysis

#### CTCF motif analysis

We downloaded the raw hESC ChIP-seq and mESC ChIP-exo data from the GEO database under accession numbers GSE69646 and GSE98671 and processed them in the same in-house pipeline described in our previous publication (Jiang et al., 2020). Briefly, reads were aligned to the hg19 and mm10 genome assemblies using Bowtie2 (v2.3.5.1) (Langmead and Salzberg, 2012) in default mode. After removing duplicate reads, multiple mapped reads, and low-quality reads, SAM files were converted into BAM format using samtools. Subsequently, peaks were called on individual replicate BAM files using MACS2 (v2.2.5) (Zhang et al., 2008) callpeak with the following parameters: --nolambda –nomodel –q 1e-5. To obtain a high-confidence peak set, only peaks that overlapped by at least 1 bp between the two replicates were retained in the downstream analysis. Finally, a total of 33,246 and 35,603 CTCF peaks were identified in hESCs and mESCs, respectively. Given the average peak size of 398 bp, we extended 200 bp upstream and downstream from each peak summit to generate a 400-bp region centered at the summit to represent each peak and merged all overlapping peak regions to generate a union set of CTCF binding sites. Motif finding was performed on those extended CTCF sites using Homer (v4.10) (Heinz et al., 2010) with the Jaspar CTCF matrix (ID: MA0139.1) and with a -log10 p value threshold of 0.25 (Chang et al., 2020; Yu et al., 2015). The findMotifsGenome.pl module of Homer was used to assign CTCF motif orientation and motif scores and to discover individual motif occurrences. A list of identified CTCF peaks and included CTCF motifs is provided in Table S2. *De novo* motif discovery was also performed to confirm the distribution of motif occurrences. However, the position weight matrix of the top *de novo* computed motif was almost identical to the known CTCF motif. Therefore, these did not substantially change any results performed using the core JASPAR motif (Khan et al., 2018). To define the control set of non-CTCF binding sites (“random”), we used the shuffleBed command in BEDTools (v2.27.1) (Quinlan and Hall, 2010) with the “-chrom -noOverlapping -excl” options to randomly permute the locations of CTCF peaks within the human and mouse genomes. The bar graphs depict the number of peaks calculated based on the shuffled peaks versus the actual peak set in each category of CTCF motif counts.

#### ChIP-seq meta-analysis

Four mESC ChIP-seq datasets (SMC1, OCT4, NANOG, CBP and Input) were downloaded from the GEO database under accession number GSE44286 and processed as described above (Whyte et al., 2013). Raw reads were aligned to the mm10 mouse reference genome using Bowtie2 with the default parameters. Peaks were called using MACS2 with the “-c” option against the input control and a p value threshold of 10^−5^ to ensure high confidence. Wiggle files representing counts of ChIP-Seq reads were created with the parameters “--nomodel –shift 200.” The resulting bigwig files were normalized for sequencing depth by dividing the read counts in 50 bp bins by the millions of mapped reads in each sample. To perform the metagene analysis, the bigwig files were quantified across each CTCF peak and its corresponding ±5 kb flanking regions using computeMatrix in deepTools (v3.4.3; Ramirez et al., 2016), and then the ChIP-seq densities in these regions were aggregated and displayed as an average profile via the plotprofile module of deepTools.

#### Functional correlation analysis

To investigate the relationship between functional insulating properties and CTCF protein binding affinity, we downloaded CTCF-CTCF loops in hESC ChIA-PET data from a previous study (Ji et al., 2016) and required at least one instance of the CTCF motif at both ends of these loops. Because CTCF-mediated chromatin loops were considered to function as insulated neighborhoods, which in turn form topologically associating domains (TADs) according to the literature, we adopted a well-known approach named the directional index, initially designed for Hi-C analysis, to calculate the loop insulating score. Briefly, each intrachromosomal ChIA-PET interaction was first mapped to a nonoverlapping 40 kb bin matrix. Each end of that PET was independently assigned to its respective bin. All pairwise bin-to-bin interaction signals were aggregated by taking the sum, thus creating a matrix of interaction frequencies between bins. Finally, insulating scores were calculated from these matrices as the log2 ratio of upstream to downstream contact frequencies for each region I at distances below 400 kb. ChIP-seq signal enrichment was computed from normalized tag densities at each CTCF binding region, representing the binding strength of that protein. Then, we grouped insulation loops with their left and right anchors into five bins from high to low based on the insulating score and analyzed the correlation between the corresponding chromatin binding and insulation levels within each category. The distribution of assigned signal values was plotted as a violin plot. The distribution of CTCF binding signals at grouped TAD anchors from mESC Hi-C data shown in Figure 3C was created in a similar fashion, as TADs are functionally equivalent to loop domains or insulated neighborhoods.

#### DNA binding domain mutation analysis

DNA-binding domains (DBDs) of sequence-specific transcription factors (TFs) were downloaded from a previously published census of human TFs (Barrera et al., 2016; Deplancke et al., 2016; Lambert et al., 2018). In total, 1,254 genes from the original list had currently valid Ensembl gene IDs and were matched to one of the DBD classes that were used for subsequent analyses. In the next step, we used variant annotations obtained from COSMIC (v87) to link amino acid substitutions to human transcription factors (Tate et al., 2019). COSMIC is a comprehensive resource for exploring the effects of somatic mutations in human cancer. It also provides a tool to map protein missense, in-frame deletion, and missense mutations to protein sequence and structure. For the present purposes, we focused strictly on functional missense variants. By mutational analysis, we aimed to characterize amino acids within the TF DBDs that might be crucial for DNA-binding and functional activity. First, DBD structural classes and the coordinates of amino acid substitutions corresponding to a specific TF were retrieved from manually curated human TF annotations. The transcript with unique gene IDs that matched the gene name to COSMIC was selected to represent the structural and mutation information for that transcription factor. To examine the distribution of amino acid substitutions in transcription factors that had DNA-binding domains and all transcription factors, we employed mutation spectrum analysis to show the relative contribution of each amino acid alteration type in those two catalogs. The bar plots depict the mean relative contribution of the top 9 amino acid substitution types over all transcription factors and DBDs of TFs. The total number of mutations in the selected case is indicated, and error bars show the standard deviation. The stacked bar length was calculated as the average relative contribution of amino acid substitution types in each cancer type. To determine the mutational spectrum of typical cancer, we stratified all TFs and TFs with DBDs into ten subpanels according to cancer types, sorted by mutation counts.

Notes on the data usage in the COSMIC dataset: The COSMIC v87 database stores mutational data from many sources, including whole-genome (Genome Screens) and whole-exome (Targeted Screens) sequence datasets. We used all COSMIC coding point mutations from targeted and genome-wide screens from the current release for hg19 because we noted that the TS data alone potentially limits our ability to observe an enrichment of mutations of introns or intergenic CTCF sites and thus are not captured in TS data. Therefore, the enrichment of such mutations in genome screens that predominantly contain TS data was considered a more suitable estimate of the genomic distribution for our study and further needs to be refined in the future as more GS data are collected.

## Data Availability

• All the data that support the findings of this study are available from the corresponding authors upon reasonable request. The raw images of the work can be found in: Mendeley Data: https://doi.org/10.17632/ssj66hy8sd.1. • This paper does not report original code. • Any additional information required to reanalyze the data reported in this paper is available from the lead contact upon request.

## Supplemental information

Document S1. Figures S1–S5

Table S1. Summary of CTCF motif counts at loop anchors in hESCs, related to Figure 3

Table S2. Summary of CTCF_DBD_Cosmic_missense mutations, related to Figure 4

Table S3. Summary of all TFs_Cosmic_missense mutations, related to Figure 6

Table S4. Summary of all TFs_DBD_Cosmic_missense Mutations, related to Figure 6

Table S5. DNA oligos used in this study, related to Figures 1–6
