# Supplementary material for: CTCF DNA-binding domain undergoes dynamic and selective protein–protein interactions
Source: iScience. 2022 Aug 24;25(9):105011. doi: 10.1016/j.isci.2022.105011 (PMC9474293; doi:10.1016/j.isci.2022.105011)
Supplement: Document S1. Figures S1–S5 [file mmc1.pdf]

iScience, Volume *25*

Supplemental information

CTCF DNA-binding domain undergoes dynamic and selective protein–protein interactions

Rong Zhou, Kai Tian, Jie Huang, Wenjia Duan, Hongye Fu, Ying Feng, Hui Wang, Yongpeng Jiang, Yuanjun Li, Rui Wang, Jiazhi Hu, Hanhui Ma, Zhi Qi, and Xiong Ji

Figure S1

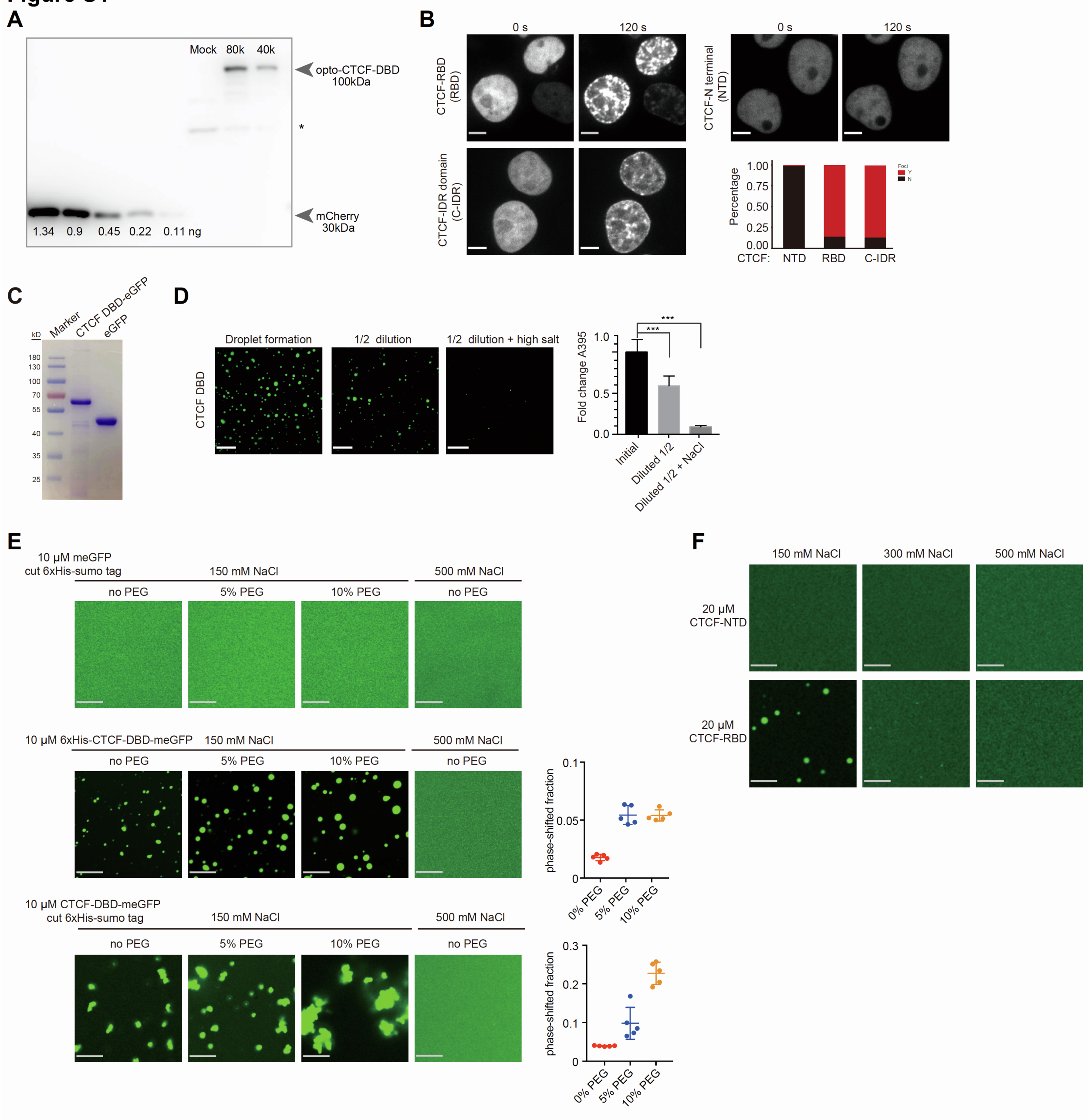

Figure S1. Self-interaction of the CTCF DBD *in vitro*, related to Figure 1.

A. Measurement of the concentration of opto-CTCF-DBD proteins. FACS-selected opto-CTCF-DBD cells were countered and western-blotted using anti-mCherry antibody, while wild-type HEK293T cells were used as the mock control (Mock). The opto-CTCF-DBD proteins were compared to serially diluted purified mCherry, and the protein concentration was calculated with two replicates. 80k and 40k indicate the cell numbers used, and "*" indicates a nonspecific band.

B. Images of HEK293T cells expressing the domains of CTCF fused to mCherry-Cry2 (opto). Representative images of light-activated cells are shown. The fluorescent proteins expressed at similar levels were activated under identical conditions. The percentages of cells in which protein clusters formed are shown in the bar graphs. Y

indicates observed clusters; N indicates no observed clusters. At least n=52 cells were used for the calculation. Scale bars, 5 μm.

C. SDS–PAGE followed by Coomassie blue staining analyses of purified recombinant CTCF DBD-EGFP and eGFP.
D. Representative images of the droplet reversibility assay. The CTCF DBD was tested with an initial buffer consisting of 10 μM protein and 150 mM NaCl at a 1:1 dilution (diluted ½) or high-salt buffer containing 500 mM NaCl. Data are represented as mean +/- SD. Scale bars, 24 μm.
E. Representative images of 10 μM meGFP, 6xHis-DBD-meGFP (monomer form of eGFP), and DBD-meGFP droplet formation in the presence of different concentrations of PEG or NaCl. Scale bar, 10 μm. Quantification of the phase-shift fraction of 6xHis-DBD-meGFP and DBD-meGFP in the presence of different concentrations of PEG. Data are represented as mean +/- SD.
F. Representative images of 20 μM CTCF NTD (top) and CTCF RBD (bottom) droplet formation in the presence of different concentrations of NaCl. Scale bars, 10 μm.

Figure S2

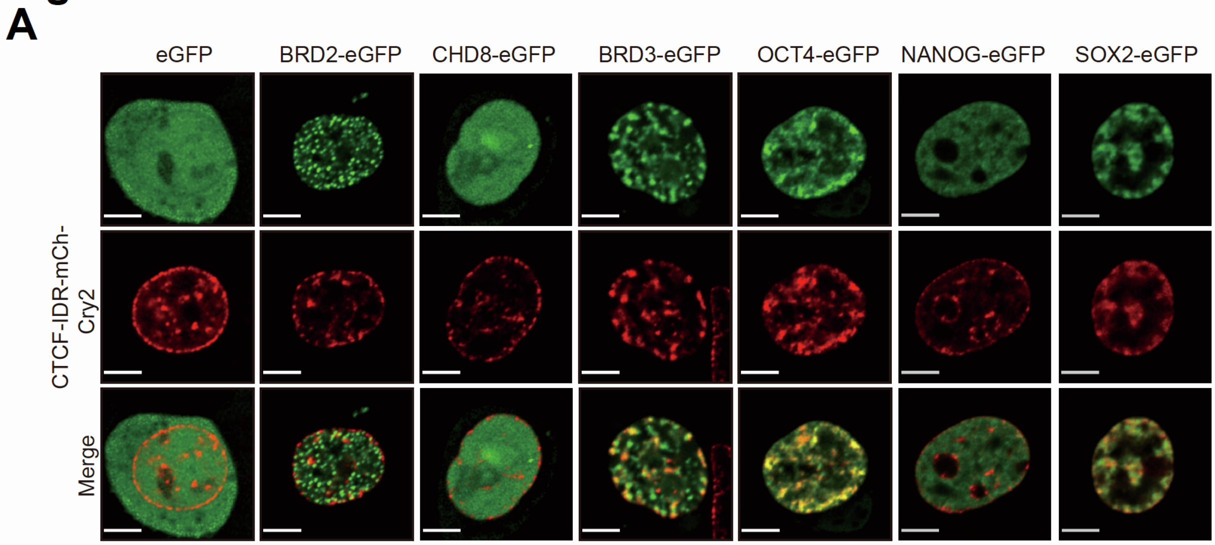

Figure S2. CTCF-IDR optoDroplets interact differently from the CTCF DBD, related to Figure 2.

A. Representative images of HEK293T cells expressing CTCF-IDR-mCh-Cry2 with eGFP, BRD2-eGFP, CHD8-eGFP, BRD3-eGFP, OCT4-eGFP, NANOG-eGFP, and SOX2-eGFP. Representative images of blue light-activated cells are shown. Scale bars, 5 μm.

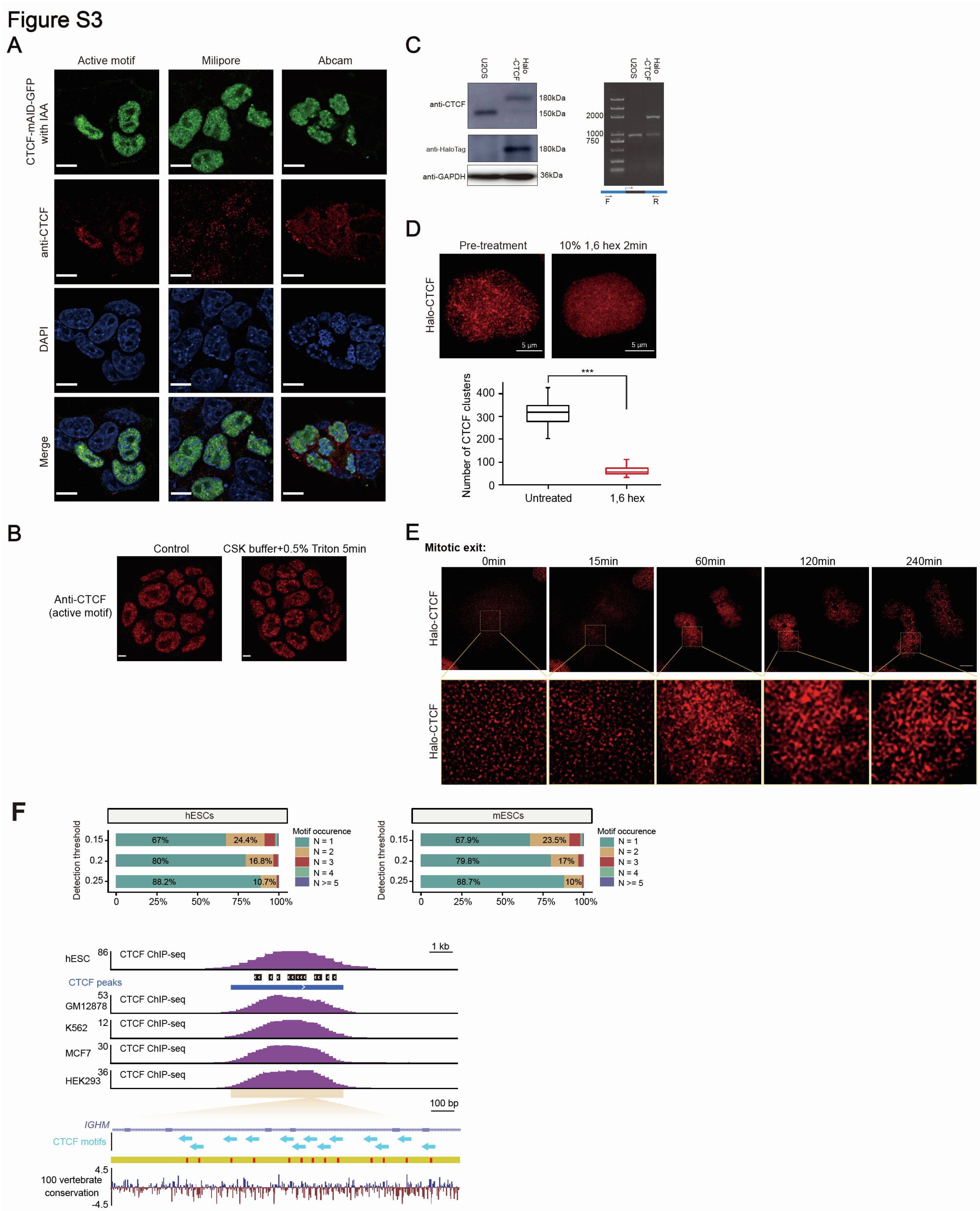

Figure S3. Endogenous CTCF forms small protein clusters, related to Figure 3.

A. Immunofluorescence (IF) images of CTCF-mAID-GFP cells obtained using different antibodies. Endogenous CTCF-mAID-GFP is shown in green, the fluorescence signal is shown in red, and DAPI staining is shown in blue at the bottom. Scale bars, 10 μm.

B. Immunofluorescence (IF) imaging of CTCF-mAID-GFP cells using different fixation protocols. Scale bars, 5 μm.
C. Immunoblotting and genotyping identification of Halo-tagged CTCF in U2OS cells are shown.
D. Representative images of halo-tagged CTCF in U2OS cells under treatment with 10% 1,6-hexanediol for 2 min. Box plot illustrating the fold change in the number of CTCF halo-tagged CTCF clusters under 1,6-hexanediol treatment (bottom). For calculation, n=68 in the control group and n=70 in the 1,6-hexanediol treatment group were used. P values were calculated using an unpaired two-tailed Student's t test (***<0.001, ****<0.0001). Scale bars, 10 μm.
E. HaloTag-CTCF U2OS cells were imaged. HaloTag-CTCF was visualized by adding 200 nM HaloTag-JF549, and cells were chosen to track the formation of CTCF clusters during mitotic exit. The cells were imaged every 15 min for 240 min. scale bar, 5 μm.
F. Upper: Stacked bars representing the percentage of occurrence frequency of CTCF motifs at different detection thresholds for CTCF peaks in hESCs and mESCs. Lower: Genome browser view of CTCF ChIP–seq signals across different cell lines around representative CTCF peaks (*IGHM*, chr14:106,762,381-106,768,280, hg19). The middle panel depicts all putative CTCF binding motifs as blue arrows, which indicate the orientation of the motif. The 100-way vertebrate conservation obtained from the UCSC genome browser is depicted at the bottom.

Figure S4

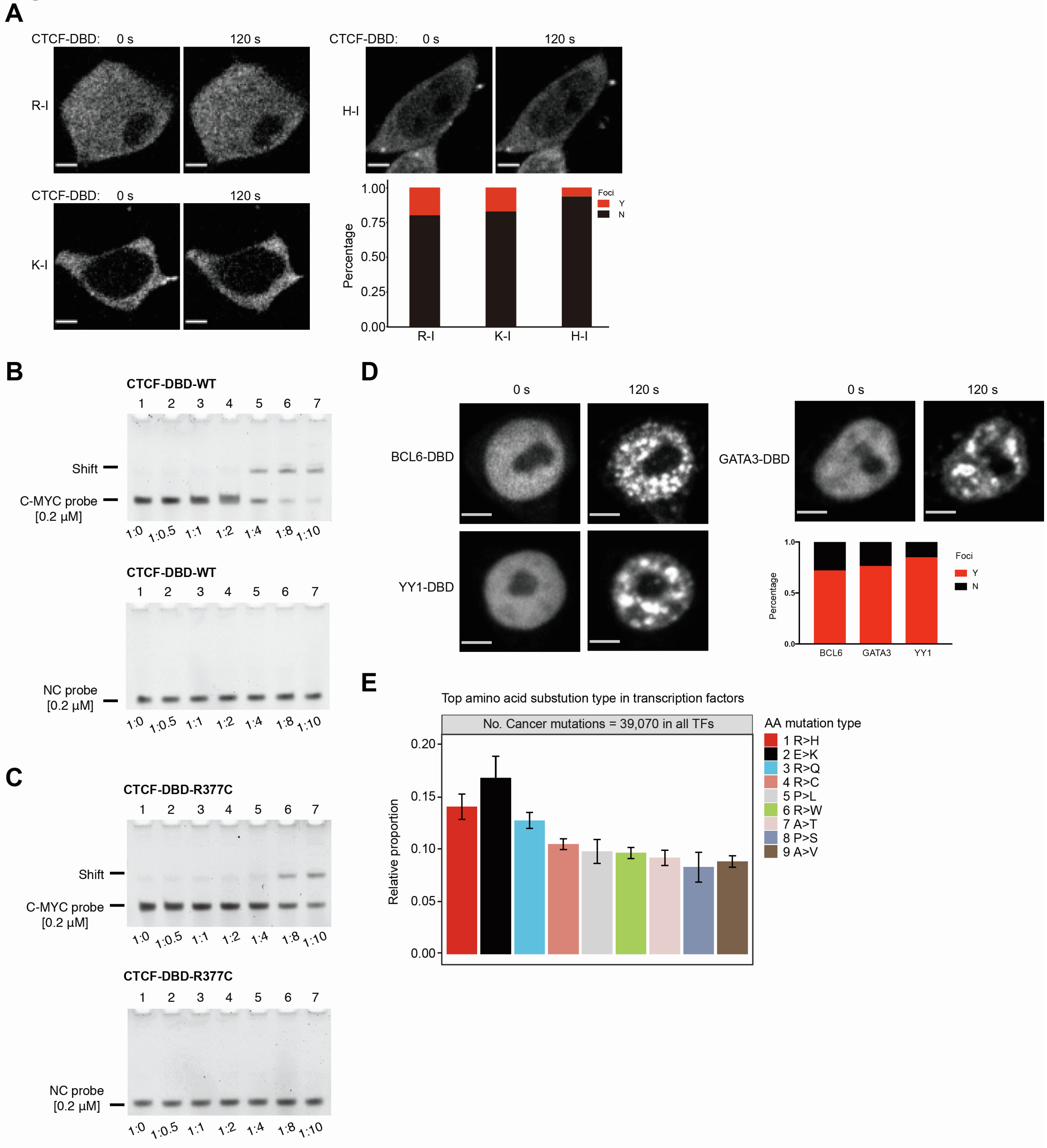

Figure S4. Arginine residues are critical for the DNA binding and self-interaction of the CTCF DBD, related to Figure 4.

A. Representative images of light-activated HEK293T cells expressing CTCF DBD-saturated mutants (R-I, H-I, and K-I, all the arginine, histidine, or lysine residues of the CTCF DNA binding domains are mutated to isoleucine) fused to mCherry-Cry2. The plotted data came from at least n=37 cells under each condition. Scale bars, 2 µm.

B. Electrophoretic mobility shift assay (EMSA). DNA (0.2 μM) (C-MYC target DNA (top) or a negative control (NC) DNA probe (bottom)) was incubated with the purified wild-type CTCF DBD and analyzed via native PAGE.
C. Electrophoretic mobility shift assay (EMSA). DNA (0.2 μM) (C-MYC target DNA (top) or a negative control (NC) DNA probe (bottom)) was incubated with purified CTCF-DBD-R377C and analyzed via native PAGE.
D. Representative images of HEK293T cells expressing the DBDs of BCL6-mCh-Cry2, YY1-mCh-Cry2 and GATA3-mCh-Cry2. Representative images of light-activated cells are shown. Scale bars, 5 μm.
E. Most frequent types of missense amino acid mutations in the transcription factors in the COSMIC database.

Figure S5
A

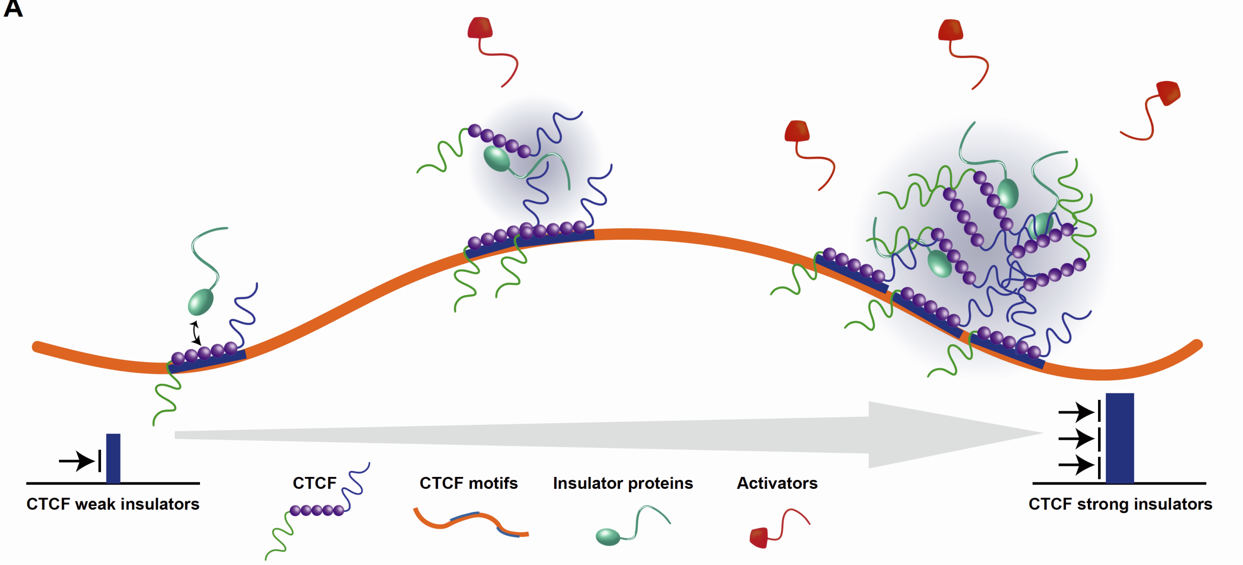

Figure S5. Spatial segregation model of CTCF insulation, related to Figures 1-5. CTCF binds its target motif, selectively interacts with insulator proteins, and preferentially avoids high densities of transcriptional activators. These properties lead to relatively high concentrations of insulator proteins and low concentrations of transcriptional activators at CTCF-bound positions, which will spatially segregate the communication of transcriptional activators between enhancers and promoters. As the binding strength of CTCF increases, its insulation capacity also increases. Note: The spatial segregation model was proposed based on the results for the CTCF DBD, which need further validation with full-length endogenous CTCF in cells in the future.
